# MBD2 facilitates tumor metastasis by mitigating DDB2 expression

**DOI:** 10.1038/s41419-023-05804-1

**Published:** 2023-05-04

**Authors:** Lei Zhang, Siyuan Wang, Guo-Rao Wu, Huihui Yue, Ruihan Dong, Shu Zhang, Qilin Yu, Ping Yang, Jianping Zhao, Huilan Zhang, Jun Yu, Xianglin Yuan, Weining Xiong, Xiangliang Yang, Tuying Yong, Cong-Yi Wang

**Affiliations:** 1 Department of Respiratory and Critical Care Medicine, The Center for Biomedical Research, NHC Key Laboratory of Respiratory Diseases, Tongji Hospital, Tongji Medical College, Huazhong University of Science and Technology, 1095 Jiefang Avenue, 430030 Wuhan, China; 2 Department of Gerontology, The Central Hospital of Wuhan, Tongji Medical College, Huazhong University of Science and Technology, Wuhan, China; 3 Department of Thoracic Surgery, Tongji Hospital, Tongji Medical College, Huazhong University of Science and Technology, 1095 Jiefang Avenue, 430030 Wuhan, China; 4 Department of Oncology, Tongji Hospital, Tongji Medical College, Huazhong University of Science and Technology, 430030 Wuhan, China; 5 Department of Respiratory and Critical Care Medicine, Shanghai Key Laboratory of Tissue Engineering, Shanghai Ninth People’s Hospital, Shanghai Jiaotong University School of Medicine, 639 Zhizaoju Lu, 200011 Shanghai, China; 6 National Engineering Research Center for Nanomedicine, College of Life Science and Technology, Huazhong University of Science and Technology, 430074 Wuhan, China

**Keywords:** Targeted therapies, Metastasis

## Abstract

Despite past extensive studies, the pathoetiologies underlying tumor metastasis remain poorly understood, which renders its treatment largely unsuccessful. The methyl-CpG-binding domain 2 (MBD2), a “reader” to interpret DNA methylome-encoded information, has been noted to be involved in the development of certain types of tumors, while its exact impact on tumor metastasis remains elusive. Herein we demonstrated that patients with LUAD metastasis were highly correlated with enhanced MBD2 expression. Therefore, knockdown of MBD2 significantly attenuated the migration and invasion of LUAD cells (A549 and H1975 cell lines) coupled with attenuated epithelial–mesenchymal transition (EMT). Moreover, similar results were observed in other types of tumor cells (B16F10). Mechanistically, MBD2 selectively bound to the methylated CpG DNA within the *DDB2* promoter, by which MBD2 repressed DDB2 expression to promote tumor metastasis. As a result, administration of *MBD2* siRNA-loaded liposomes remarkably suppressed EMT along with attenuated tumor metastasis in the B16F10 tumor-bearing mice. Collectively, our study indicates that MBD2 could be a promising prognostic marker for tumor metastasis, while administration of *MBD2* siRNA-loaded liposomes could be a viable therapeutic approach against tumor metastasis in clinical settings.

## Introduction

Tumor metastasis is featured by the dissemination of tumor cells to new sites, leading to the formation of secondary tumors, which has been under intensive investigations for preventing distant tumor spread and related mortality [1]. During the course of metastasis, malignant cells firstly become migratory and invasive to spread them from the primary tumor, by which they get into the circulation *via* blood vessel or lymphatic intravasation [2]. Approximately 0.2% of the circulating tumor cells (CTCs) could evade immune surveillance and infiltrate into the vascular system to initiate distant metastatic colonization. However, the disseminated tumor cells require a new microenvironment in favor of the establishment of a metastatic supportive niche at distant sites, where they convert from the migratory mode to the proliferative mode, thereby inducing the large-scale spread [3].

Accumulating evidence suggests that epithelial–mesenchymal transition (EMT) plays a central role in tumor metastasis [4]. EMT is an important morphological change that entails the transformation of epithelial cells into an elongated fibroblastic phenotype. The process is characterized by the downregulation of epithelial markers (e.g., E-cadherin) and upregulation of mesenchymal markers (e.g., N-cadherin and Vimentin) following the stimulation of specific growth factors, in which the transforming growth factor beta1 (TGF-β1) is the prototype [5, 6]. Cancer cells undergone EMT display increased motility and invasiveness to promote their dissemination to the remote spots [6]. Indeed, TGF-β1 activates its downstream signaling pathways and several transcription factors that are essential for EMT process [7]. Therefore, targeting TGF-β1 has been proved to be an efficient therapeutic strategy for repressing tumor metastasis [8]. However, the adverse effects are even worse than the therapeutic impacts, which limited its clinical application.

DNA methylation is a chemical epigenetic modification essential for cell viability and embryonic development in mammals. Recent studies demonstrated evidence supporting that DNA methylation also plays a crucial role in EMT process [9]. Given that global inhibition of DNA methylation is always coupled with severe side effects, strategies aimed at suppressing DNA methylation cannot be applied in clinical settings. However, studies including our own revealed that the information encoded by DNA methylome is read by a family of methyl-CpG–binding domain (MBD) proteins such as MBD1 to 4, and the methyl CpG binding protein 2 (MeCP2) [10]. MBD1, MBD2, MBD4, and MeCP2 selectively bind to the methylated CpG DNA to suppress target gene transcription *via* a transcriptional repressor domain (TRD). In particular, MBD2 possesses the highest binding activity [11], but mice deficient in *MBD2* is viable and fertile without perceptible defects in physiological activities [12]. Therefore, MBD2 could be a viable target against tumor metastasis without discernable side effect. Indeed, altered MBD2 expression has been consistently noted to impact tumorigenesis on mouse cancer cell lines and on human cancer cell xenograft models [13], but its impact on tumor metastasis is yet to be elucidated.

Herein in this report, we demonstrated convincing evidence that patients with lung adenocarcinoma (LUAD) at the stage of lymphatic or distant metastasis were featured by the enhanced MBD2 expression. Moreover, knockdown of MBD2 significantly attenuated TGF-β1-induced EMT in LUAD cell lines (A549 and H1975) as evidenced by the repressed migration and invasion. Interestingly, consistent results were also observed in murine melanoma B16F10 cells, suggesting that altered MBD2 expression is a common feature adopted by tumor cells for metastasis. Mechanistically, MBD2 selectively bound to the methylated CpG DNA within the damage-specific DNA binding protein 2 (*DDB2*) promoter, by which it repressed DDB2 expression to enhance EMT process, thereby leading to tumor metastasis. As a result, liposomes loaded with *MBD2* siRNA suppressed tumor metastasis in a B16F10 tumor-bearing mouse model. Together, our data support that MBD2 could be a promising prognostic marker for tumor metastasis, while strategies aimed at silencing MBD2 with siRNA-loaded liposomes could be a viable approach against tumor metastasis in clinical settings.

## Methods

### Tissue samples

Fresh tissue samples (138 in total) including lung adenocarcinoma (LUAD) (*n* = 69) and paired adjacent normal lung (*n* = 69, >5 cm from the tumor edge) tissues, were collected from Tongji Hospital, Tongji Medical College, Huazhong University of Science and Technology, Wuhan, China. All patients had received no chemotherapy or radiotherapy before surgery and signed a written informed consent before sample collection. Clinical data of the patients are provided in Supplementary Table 1. The studies were approved by the Human Assurance Committee of Tongji Hospital (IRB: TJ-IRB20160601).

### Data mining from TCGA and Human Protein Atlas database

We assessed the association between DDB2 expression and melanoma using the cohorts from TCGA (The Cancer Genome Atlas) database (TCGA-melanoma cohort). The survival curves for MBD2 and DDB2 in LUAD and melanoma patients were evaluated in the data from the Human Protein Atlas database (https://www.proteinatlas.org/). The UCSC Xena browser (https://xenabrowser.net) was employed to access and analyze the data.

### Animals

The C57BL/6 male mice (8 weeks old) were purchased from Beijing Vital River Laboratory Animal Technology Co., Ltd. (Beijing, China). All animal experiments were conducted in accordance with the guidelines approved by the Institutional Animal Care and Use Committee at Tongji Hospital, Tongji Medical College, Huazhong University of Science and Technology (Wuhan, China).

### Cell culture

The A549 cells, NCI-H1975 and murine melanoma B16F10 cells were purchased from the American Type Culture Collection (Rockville, MD, USA). A Mycoplasma contamination test was carried out, which was negative. Those cells were cultured at 37 °C in a 5% CO_2_ incubator. To stimulate EMT, each type of cells was induced by TGF-β1 (10 ng/ml, 24 h) after transfected with *MBD2* siRNA or a scramble siRNA for 48 h. The scramble siRNA transfected cells without TGF-β1 were served as the controls.

### Reagents

Recombinant human TGF-β1 (Cat: 100-21) was purchased from the PeproTech Corporation (Cranbury, NJ, USA). Antibodies against E-cadherin (Cat: Sc-7870), N-cadherin (Cat: Sc-8424), Vimentin (sc-58899) and GAPDH(Sc-47724) were obtained from the Santa Cruz Biotechnology (Dallas, TX, USA). The crystal violet staining solution (Cat: C0121) and the ChIP assay kit (Cat: P2078) were purchased from the Beyotime Biotechnology (Shanghai, China). The lipidoid C12-200 was purchased from the Xinjiahecheng Medical Chemistry Corporation (Wuhan, Hubei, China). 1,2-Dimyristoyl-rac-glycero-3-methoxypolyethylene glycol-2000 (mPEG-DMG) was ordered from the NOF Corporation (Tokyo, Japan). The CCK-8 assay kit (Cat: 40203ES60) was originated from Yeasen Biotechnology (Wuhan, China), and the Lipofectamine 3000 (Cat: L3000015) was obtained from the Invitrogen Corporation (Shanghai, China). All other reagents were obtained from Sigma (St. Louis, MO, USA).

### RNA interference and preparation of siRNA-loaded liposomes

The *MBD2* siRNAs and a corresponding scramble siRNA were synthesized by the RiboBio (Guangzhou, China), and were then transfected into A549 cells, B16F10 cells, and H1975 cells using the Lipofectamine 3000 according to the manufacturer’s protocol or a liposome-based siRNA transfection method as previously described [14]. The following two siRNAs were used for MBD2: siRNA1 5’-GCA AGA GCG AUG UCU ACU A-3’, and siRNA2 5’-GCG AAA CGA UCC UCU CAA U-3’. The siRNA-loaded nanoparticles were prepared as reported [15].

### MBD2 overexpression

A549 cells and B16F10 cells were seeded on 12-well plates and transduced with Adenovirus-*MBD2* and a Vector (Abcam, MA, USA) at the multiplicity of infection (MOI) 1:4 for 48 h. The transduced cells were then stimulated with TGF-β1 (10 ng/ml) for 24 h and analyzed by Western blotting.

### Western blot analysis

Total proteins were extracted from tissues and cells using the Lysis buffer (Beyotime, Shanghai, China), and then subjected to Western blot analysis using the established techniques [16]. The primary antibodies against MBD2; E-cadherin, N-cadherin and Vimentin were employed for the analyses, and GAPDH band was used as the internal control.

### Immunofluorescence assay

Cells were fixed with 4% paraformaldehyde for 10 min and permeabilized with 0.1% Triton X-100 for 15 min. The samples were washed with PBS and blocked with 5% BSA in PBS for 1 h. Subsequently, the cells were incubated firstly with indicated primary antibodies overnight at 4 °C, and then with an Alexa Fluor 488-conjugated antibody or an Alexa Fluor 594-conjugated antibody (Abbkine, Redlands, CA, USA, 1:200) for 1 h at room temperature, respectively. Nuclei were counterstained with DAPI for 8 min. Immunofluorescence images were acquired under a microscope (Olympus, Tokyo, Japan) in a blinded manner.

### Quantitative real-time PCR

Quantitative real-time PCR was conducted using the SYBR Premix Ex Taq (TaKaRa, Tokyo, Japan) as previously reported [17]. The primer sequences were provided in Supplementary Table 2.

### Cell migration and invasion assays

The cell migration and invasion were evaluated by using a Transwell assays (Corning, MA, USA) using the established techniques [18].

### Cell Counting Kit-8 Assay

The cell viability was examined by a Cell Counting Kit-8 kit based on the manufacturer’s protocol. The absorbance was measured at 450 nm using a microplate reader (ELx800, BioTek Instruments, Winooski, VT, USA).

### Apoptosis assay

Apoptosis assay was conducted using an Annexin V/PI detection kit (Beyotime, Shanghai, China) and analyzed by flow cytometry (BD Biosciences, San Jose, CA, USA) as reported previously [18].

### RNA deep sequencing (RNA-seq)

RNA from A549 cells activated by TGF-β1 was extracted using an RNA isolation kit (TaKaRa, Tokyo, Japan). The prepared mRNA libraries were sequenced on an Illumina HISEQ 2500 platform, and the HISAT2 v2.0.4 software (Center for Computational Biology, Baltimore, Maryland) was used to map the clean reads to the mm10 reference genome. Fragments per kilobase of exon per million mapped fragments (FPKM) values were calculated using the CuffNorm version 2.2.1 software (University of Washington, Seattle, Washington, USA). The genes with a calculated normalized FPKM value greater than 5.0 were considered to be expressed. Significantly differentially expressed genes were defined as those with a |log2 (fold change)| ≥ 0 and a *p* value ≤ 0.05. Heatmaps were generated using the R package heatmap.

### GO term and KEGG pathway enrichment analysis

The biological significance of the differentially expressed genes (DEGs) was explored by the Gene Ontology (GO) term enrichment analysis covering 3 functional groups (biological process, cellular component and molecular function), and the Genomes (KEGG) pathway enrichment analysis of the DEGs was performed with the Bioconductor package “GeneAnswers” to identify vital pathways related to EMT in A549 cells.

### Global DNA methylation assay and bisulfite DNA sequencing

Global DNA methylation was evaluated by a MethylFlashTM Methylated DNA Quantification Kit (Epigentek, NY, USA) according to the instructions and bisulfite DNA sequencing was carried by Sequenom MassARRAY platform (BGI.write, Beijing, China) as previously reported [19].

### Chromatin immunoprecipitation (ChIP) assay and DDB2 promoter reporter assay

ChIP assays were conducted using a ChIP assay kit (Beyotime, Shanghai, China) as reported [20]. The primers for *DDB2* used in the ChIP PCR were F: 5’ ACT CCC CAA CTA CAC CCT GT 3’ and R: 5’ CCG GCT AAT TTC TCT CTC TCT 3’. A dual luciferase reporter system (Promega, Madison, WI) was used for DNA methylation dependent *DDB2* promoter luciferase reporter assays, in which the MBD2 binding sites within the *DDB2* promoter were disrupted using the established techniques [21].

### Flow cytometric analysis

A549 and B16F10 cells were transfected with DiI-labeled liposomes for 2 or 4 h. After washing, the cells were analyzed using a FACSCanto II (BD Biosciences, San Jose, CA, USA) as reported [22]. All data were analyzed using the FACSExpress V3 software (De Novo Software) based on the manufacturer’s protocol.

### Anticancer activity in the B16F10 lung metastasis model

The melanoma tumor-bearing mouse model was established through the subcutaneous injection of B16F10 melanoma cells into the right flanks of C57BL/6 mice (6 ×10^5^ cells/mouse). Once the tumor volume reached approximately 80 mm^3^, the mice were randomly intratumorally injected with PBS, L-scramble siRNA, empty liposomes or L-*MBD2* siRNA (1 mg/kg) for a total of three times with a three-day of interval, eight mice were included in each study group. Tumor progression was assessed by monitoring the tumor volume with the following equation: *V* = (length × width^2^)/2. Two days after the last injection, the mice were sacrificed, and the tumor tissues were collected and weighed. The tumors were cryosectioned into pieces. The sections were co-stained with a FITC-labeled anti-N-cadherin antibody and an Alexa Fluor 594-labeled anti-E-cadherin antibody at 37 °C for 30 min. After rinsing the sections with PBS. the distribution of N-cadherin and E-cadherin was observed by confocal microscopy, and the relative fluorescence intensity was determined using the ImageJ software.

To further investigate the inhibitory capability of L-*MBD2* siRNA in tumor metastasis, B16F10 melanoma cells were incubated with PBS, empty liposomes, 50 nM L-scramble siRNA or 50 nM L-*MBD2* siRNA for 6 h, respectively. The pretreated B16-F10 tumor cells were then intravenously injected into the C57BL/6 mice (2 × 10^5^ cells/mouse). After 20 days of injection, metastatic tumor-bearing lung tissues were collected from sacrificed mice, and the pulmonary metastatic nodules were counted. Tumor-bearing lung sections were also prepared for H&E staining.

### Statistical analysis

All experiments were conducted with at least three independent replicates, and statistical analyses were performed using the GraphPad Prism 5.0 software (San Diego, CA, USA). Data are presented as the mean ± SEM, and an independent Student’s *t* test was applied to analyze the statistical significance of differences between two groups. As for comparisons among multiple groups, one-way ANOVA followed by Tukey’s post hoc test was performed. In all cases *p* < 0.05 were considered with statistical significance.

## Results

### LUAD metastasis is coupled with MBD2 upregulation

**Fig. 1:**
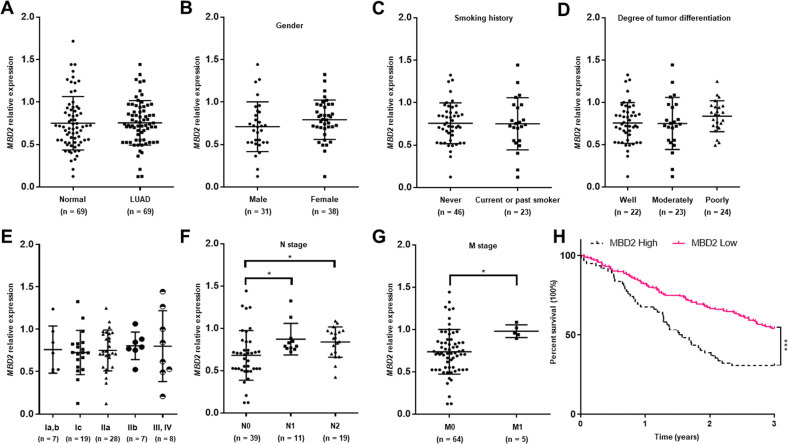
Expression of MBD2 and its clinical significance in lung adenocarcinoma patients. **A** The mRNA level of MBD2 was compared between 69 pairs of lung adenocarcinoma tissue samples (LUAD) and adjacent nontumor tissue samples (Normal). The expression of MBD2 was associated with sex (**B**), smoking history (**C**), degree of tumor differentiation (**D**), tumor stage (**E**), N stage (**F**), and M stage (**G**) in lung adenocarcinoma patients (*n* = 69). **H** Survival curves for MBD2 high group and MBD2 low group (The cutoff value is 15.02, *n* = 269). The data were originated from the Human Protein Atlas database. Each dot expresses one patient sample. N node, M metastasis. The data are presented as the mean ± SD. *p < 0.05.

Since lung cancers ((non-small cell lung cancers (NSCLCs) in particular) are one of the highest-ranking causes of cancer-related death worldwide and LUAD is the most prevalent subtype of NSCLCs [23], we thus first examined *MBD2* expression in the lung samples obtained from 69 LUAD patients. No significant difference in terms of *MBD2* mRNA was observed between the adjacent normal tissue samples and the LUAD tissue samples (Fig. 1A). A similar expression pattern was also found in LUAD tumor tissues (*n* = 58) versus matched adjacent nontumor tissues (*n* = 58) [24] derived from the Oncomine database (https://www.oncomine.org/resource/login.html) (Supplementary Fig. S1A, B), a publicly accessible online cancer microarray database.

To explore the functional impact of MBD2 on LUAD, we evaluated the associations between MBD2 expression and the clinicopathological features of LUAD patients. First, we observed that *MBD2* expression in tumors was not correlated with sex (Fig. 1B), smoking history (Fig. 1C), or the degree of tumor differentiation (Fig. 1D). Similarly, we failed to detect a significant difference of *MBD2* expression between tumors at different tumor stages (Fig. 1E). In sharp contrast, tumors with lymphatic or distant metastasis exhibited significantly higher *MBD2* expression than that of tumors in situ (Fig. 1F, G). Moreover, high levels of MBD2 expression were found to be associated with poor outcome in patients with LUAD derived from the Human Protein Atlas database (Fig. 1H). To further confirm the above results, we examined our collected LUAD samples. Collectively, these data support that MBD2 is likely involved in tumor metastasis during the course of LUAD progression.

### MBD2 regulates tumor metastasis in vitro

**Fig. 2:**
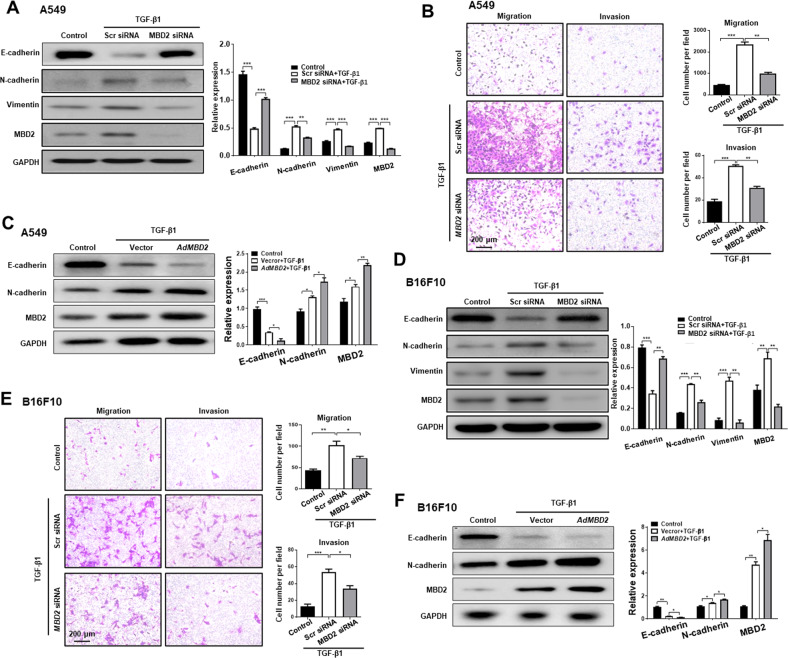
Silencing of MBD2 inhibited TGF-β1-induced cell migration, invasion and EMT. **A** Western blot analysis of E-cadherin, N-cadherin, Vimentin and MBD2 in A549 cells after *MBD2* siRNA transfected. **B** Transwell assays were performed to investigate the migration ability (left panel) and invasion ability (right panel) of A549 cells. **C** Western blot analysis of E-cadherin, N-cadherin and MBD2 in A549 cells after Ad*MBD2* transduced. **D** Western blot analysis of E-cadherin, N-cadherin, Vimentin and MBD2 in B16F10 cells after *MBD2* siRNA transfected. **E** Transwell assays were performed to investigate the migration ability (left panel) and invasion ability (right panel) of B16F10 cells. **F** Western blot analysis of E-cadherin, N-cadherin and MBD2 in B16F10 cells after Ad*MBD2* transduced. All images were acquired at ×200 magnification. The data are presented as the mean ± SEM. *p < 0.05, **p < 0.01, ***p < 0.001.

To confirm the above observations in LUAD patients, we next conducted Transwell assays to assess the impact of MBD2 on the migration and invasion of multiple cancer cell lines. To this end, A549 cells, a NSCLC cell line, were transfected with a scramble siRNA or an *MBD2* siRNA for 48 h with Lipofectamine 3000 and then stimulated with TGF-β1 for 24 h, respectively. Notably, Western blot analysis revealed that TGF-β1 induced a significant increase of MBD2 expression, which was robustly blocked by the *MBD2* siRNA (Fig. 2A), indicating that the *MBD2* siRNA possesses high potency to knock down MBD2 expression. Importantly, TGF-β1 induced a dramatic increase in the number of migrated and invaded cells as compared to those of control cells, while knockdown of MBD2 potently reversed TGF-β1-induced migration and invasion (Fig. 2B). These results prompted us to examine the expression of E-cadherin, N-cadherin and Vimentin, the critical EMT markers relevant to tumor metastasis [25]. As expected, TGF-β1 robustly repressed E-cadherin expression, but promoted N-cadherin and Vimentin expression (Fig. 2A). Remarkably, the *MBD2* siRNA significantly blocked the effect of TGF-β1 on the induction of EMT process (Fig. 2A and Supplementary Fig. S2A). Next, we checked morphological changes of the above cells by FITC-phalloidin staining. It was noted that A549 cells treated with TGF-β1 displayed an elongated spindle-like morphology, while significantly less amount of spindle-shaped cells was noted in MBD2 siRNA transfected cells (Supplementary Fig. S2B), suggesting that knockdown of MBD2 may attenuate TGF-β1-induced EMT in A549 cells.

To address whether the above findings were limited to A549 cells, we conducted studies in murine melanoma B16F10 cells, which is generally considered as a potent metastatic tumor cell line. Similar results were noted as above in B16F10 cells, and *MBD2* siRNA substantially repressed TGF-β1-induced MBD2 expression (Fig. 2D), and the *MBD2* siRNA exhibited high potency to suppress TGF-β1-induced migration, invasion (Fig. 2E) coupled with attenuated EMT, as evidenced by the changes of expression for EMT related makers (Fig. 2D and Supplementary Fig. S2C) and cellular morphology (Supplementary Fig. S2D). Consistent results were obtained from in parallel experiments conducted in LUAD NCI-H1975 cells (Supplementary Fig. S2E). The next key question is whether MBD2 overexpression would promote EMT. To address this issue, adenoviruses carrying MBD2 cDNA were transduced into A549 and B16F10 cells. Indeed, the Ad*MBD2*-transduced cells manifested a significantly increased EMT program (Fig. 2C, F). Taken together, our data support that MBD2 enhances EMT, by which it promotes cancer cell migration and invasion predisposing to tumor metastasis.

### MBD2 represses *DDB2* transcription to enhance EMT

**Fig. 3:**
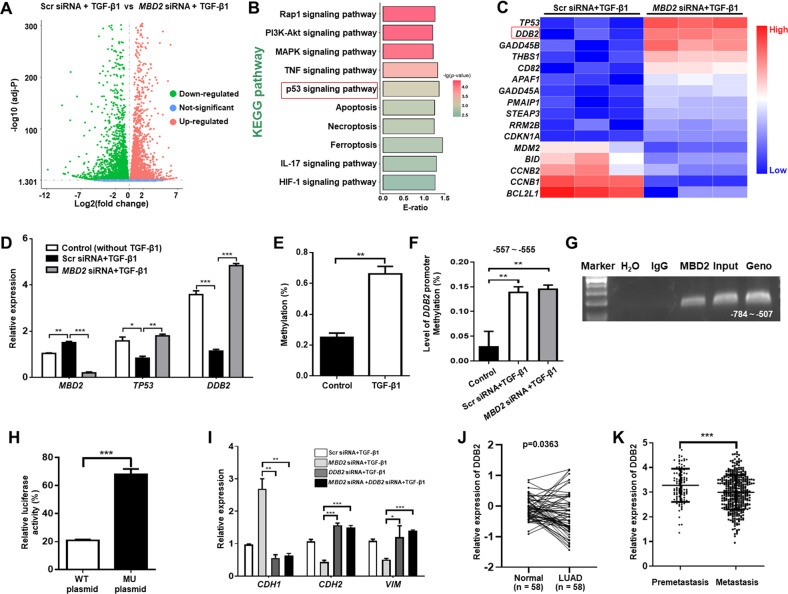
MBD2 regulates *DDB2* expression in A549 cells on TGF-β1 stimulation. **A** Scatter plot showing the average gene expression level (log10) in A549 cells upon TGF-β1 stimulation vs the fold change (log2). The blue and red dots represent downregulated and upregulated genes, respectively. **B** KEGG pathway enrichment analysis of the upregulated DEGs. **C** Heatmap showing the upregulated P53-related genes in TGF-β1-treated A549 cells transfected with *MBD2* siRNA. **D** RT-PCR analysis of *MBD2*, *TP53* and *DDB2* expression in TGF-β1 induced A549 cells after *MBD2* siRNA transfected. **E** Global DNA methylation level in A549 cells following TGF-β1 treatment. **F** Results of the bisulfite DNA sequencing analysis of the *DDB2* promoter in TGF-β1 induced A549 cells following *MBD2* siRNA transfected. **G** ChIP-PCR analysis of MBD2 binding to the *DDB2* promoter. **H** Relative luciferase activity in A549 cells after WT or MU plasmid transfected. **I** RT-PCR analysis of *CDH1*, *CDH2* and *VIM* expression in A549 cells. **J** Comparison of *DDB2* mRNA expression between lung adenocarcinoma tissue samples and adjacent nontumor tissue samples in the TCGA-LUAD cohort. **K** Comparison of *DDB2* mRNA expression between premetastatic and metastatic tissues in the TCGA melanoma cohort. The data are presented as the mean ± SEM. GO Gene Ontology, DEG differentially expressed gene, KEGG Kyoto Encyclopedia of Genes and Genomes. *p < 0.05, **p < 0.01, ***p < 0.001.

To dissect the mechanisms by which MBD2 facilitates tumor metastasis, RNA deep sequencing was conducted in scramble and *MBD2* siRNA transfected A549 cells following TGF-β1 stimulation. Comparative analysis revealed that the *MBD2* siRNA upregulated the expression of 4386 genes but downregulated the expression of 4559 genes (Fig. 3A). GO enrichment analysis characterized top 10 terms enriched with the differential expressed genes (DEGs) which involved in cell morphogenesis, adhesion and migration, indicating a pivotal role of MBD2 in tumor metastasis (Supplementary Fig. S3). KEGG pathway analysis revealed 10 pathways enriched with the upregulated genes (Fig. 3B), among which P53 signaling pathway was the most significant one under the comprehensive consideration of the enrichment ratio and *p*-value. Given that P53 is one of the most important tumor suppressor genes frequently mutated in human cancers [26], we then picked out P53-related genes from DEG and listed them in the heat map (Fig. 3C). Indeed, RT-PCR confirmed that *MBD2* siRNA enhanced *TP53* expression following TGF-β1 induction (Fig. 3D). Interestingly, the damaged DNA binding 2 (DDB2), a well-known and potent inhibitor for tumor metastasis [27], was characterized in the cluster of genes whose expression was upregulated in the *MBD2* siRNA transfected cells (Fig. 3D). Specifically, the *MBD2* siRNA rendered the TGF-β1 stimulated cells with a 3-fold increase of *DDB2* expression as compared to the scramble siRNA transfected cells (Fig. 3D), suggesting that DDB2 could be the critical MBD2 target gene. Since MBD2 serves as a reader to interpret the information encoded by DNA methylation [13], we analyzed the DNA methylation status of genomic DNA by a global DNA methylation assay. Remarkably, TGF-β1 induced a global genomic DNA hypermethylation in A549 cells (Fig. 3E), while bioinformatic analysis using *EMBOSS Cpgplot* (http://www.ebi.ac.uk/Tools/seqstats/emboss_cpgplot/) revealed that the *DDB2* promoter sequence contained a CpG island (Supplementary Fig. S4A). This result prompted us to assess the DNA methylation status of the *DDB2* promoter by bisulfite DNA sequencing. Indeed, TGF-β1 induced the CpG DNA within the *DDB2* promoter (Supplementary Fig. S4B, from −555 to −557 bp, the transcription start site as +1) to undergo a DNA hypermethylation. However, no perceptible difference in terms of DNA methylation within the *DDB2* promoter was noted between the scramble siRNA and *MBD2* siRNA transfected A549 cells, indicating that MBD2 itself does not DNA methylation (Fig. 3F).

We next sought to address whether MBD2 selectively binds to the above hypermethylated CpG DNA within the *DDB2* promoter. Chromatin immunoprecipitation (ChIP) assays were employed to determine the MBD2 binding activity to the *DDB2* promoter in A549 cells. Indeed, MBD2 could bind to the methylated CpG DNA within the *DDB2* promoter following TGF-β1 stimulation (Fig. 3G). To dissect the effect of MBD2 on DDB2 transcription following binding to its promoter, a pGL3-Basic luciferase reporter plasmid, in which the cytosine within the CpG DNA of *DDB2* promoter (from −555 to −557 bp) were mutated to adenine, was constructed (Mutant plasmid). A pGL3-Basic plasmid containing the wild-type *DDB2* promoter sequence was also prepared (WT plasmid). The A549 cells were co-transfected with either the mutant or WT luciferase reporter plasmid along with an *MBD2* plasmid, and the cells were harvested for assays of reporter activity following TGF-β1 stimulation. In line with the above results, cells transfected with the mutant plasmid displayed a 2.5-fold higher reporter activities (Fig. 3H), indicating that MBD2 represses DDB2 expression following binding to the methylated DNA within its promoter. We then embarked on the effect of DDB2 on TGF-β1 induced EMT program. A549 cells were transfected with an *MBD2* siRNA or a *DDB2* siRNA alone or in the presence of TGF-β1 stimulation. Similar as our previous data, knockdown of *MBD2* significantly increased *CDH1* expression while repressed *CDH2* and *VIM* expression (Fig. 3I). However, knockdown of DDB2 completely abolished the effect of *MBD2* siRNA, and particularly, no perceptible difference was noted in terms of the expression levels for the above genes between *DDB2* siRNA and *MBD2* + *DDB2* siRNA transfected cells (Fig. 3I). These results support that MBD2 enhances TGF-β1-induced EMT dependent on its suppressive effect on DDB2 expression.

Based on the above data, we next examined the association between MBD2 and DDB2 expression in lung tissues from LUAD patients. We first compared *DDB2* mRNA levels between Normal and LUAD in the TCGA-LUAD cohort. Analysis of the paired samples indicated that LUAD was featured by the decreased *DDB2* mRNA levels (Fig. 3J). More importantly, the *DDB2* mRNA levels were substantially lower in samples derived from metastatic tissue than that of samples originated from premetastatic tissue in the TCGA-melanoma cohort (Fig. 3K). To investigate the prognostic values of DDB2 in patients with LAUD or melanoma, the Human Protein Atlas was employed to assess the relationship between DDB2 and prognosis of tumor patients. A total of 267 LUAD patients and 47 melanoma patients were available for the analysis of overall survival. The survival curves indicated that lower levels of DDB2 expression were associated with shorter overall survival (Supplementary Fig. S5A, B), and this association is consistent with what we found for MBD2 (Fig. 1H). Collectively, our data support that MBD2 promotes tumor metastasis by enhancing EMT *via* attenuating DDB2 expression.

### Generation of *MBD2* siRNA-loaded liposomes

To translate the above discoveries into a clinical strategy against tumor metastasis, we generated cationic lipid-based liposomes carrying *MBD2* siRNA as previously reported [21] (Supplementary Fig. S6A). The prepared liposomes dispersed well in PBS (polydispersity index 0.15) and possessed an *MBD2* siRNA entrapment efficiency above 95% with a zeta potential of 2.3 mV (Supplementary Fig. S6B). The hydrodynamic diameter of these liposomes was approximately 98 nm (Supplementary Fig. S6B), which was smaller than that of the empty liposomes likely due to the electronegativity of siRNA. Transmission electron microscopy demonstrated that the liposomes exhibited a uniform distribution of spherical particles (Supplementary Fig. S6C, D) and could maintain its intact structure in PBS for at least 24 h (Supplementary Fig. S6E).

**Fig. 4:**
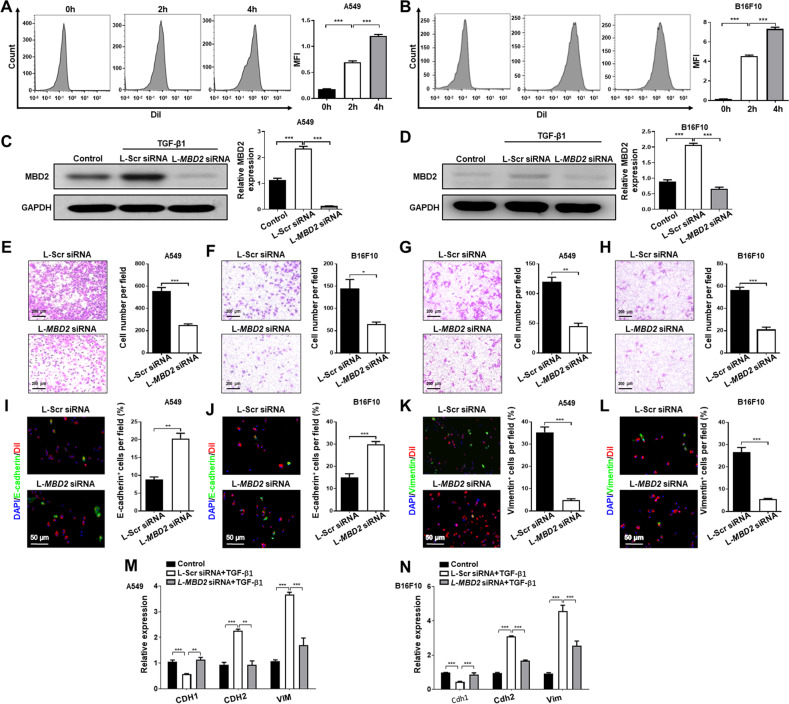
*MBD2* siRNA-loaded liposomes inhibited tumor metastasis in vitro. MIF in A549 (**A**) and B16F10 (**B**) cells transfected with DiI-labeled liposomes. **C**, **D** Western blot analysis of MBD2 expression in A549 (**C**) and B16F10 (**D**) cells after L-Scr siRNA or L-*MBD2* siRNA transfected. **E**, **F** The migration ability of A549 (**E**) and B16F10 (**F**) cells transfected with L-Scr siRNA or L-*MBD2* siRNA. **G-H** The invasion ability of A549 (**G**) and B16F10 (**H**) cells transfected with L-Scr siRNA or L-*MBD2* siRNA. **I**, **J** Coimmunostaining of liposomes (Red) and E-cadherin (Green) in A549 (**I**) and B16F10 (**J**) cells after TGF-β1 stimulation. **K**, **L** Coimmunostaining of liposomes (Red) and Vimentin (Green) in A549 (**K**) and B16F10 (**L**) cells after TGF-β1 stimulation. All images were acquired at ×200 magnification. Nuclei were stained with DAPI (blue). **M**, **N** RT-PCR analysis of *CDH1*, *CDH2* and *VIM* in TGF-β1 induced A549 (**M**) and B16F10 (**N**) cells following L-Scr siRNA or L-*MBD2* siRNA treatment. MFI mean fluorescence intensity, L-Scr siRNA liposome-based Scrambled siRNA, L-*MBD2* siRNA liposome-based *MBD2* siRNA. The data are presented as the mean ± SEM. *p < 0.05, **p < 0.01, ***p < 0.001.

Next, we assessed the toxicity of liposome-loaded siRNA by CCK-8 assay. As expected, the safety profile of those liposomes was promising, as we failed to detect any toxicity of liposome-loaded scramble siRNA in both A549 and B16F10 cells, in terms of viability, at the different concentrations tested (Supplementary Fig. S6F). In contrast, administration of *MBD2* siRNA-loaded liposomes strikingly decreased the viability of A549 and B16F10 cells once the dose of liposomes was higher than 200 nM (Supplementary Fig. S6G), while 200 nM of *MBD2* siRNA-loaded liposomes only rendered less than 10% cells to undergo apoptosis (Supplementary Fig. S6H), and therefore, this concentration was chosen for the subsequent studies. Flow cytometry analysis revealed that those liposomes could be efficiently taken up by A549 (Fig. 4A) and B16F10 cells in a time-dependent manner (Fig. 4B). A549 and B16F10 cells were then co-cultured with siRNA-loaded liposomes for 48 h and then stimulated with TGF-β1 for 24 h. The *MBD2* siRNA-loaded liposomes (L-*MBD2* siRNA) significantly repressed TGF-β1-induced MBD2 expression (Fig. 4C, D) along with attenuated migration (Fig. 4E, F) and invasion (Fig. 4G, H). Wound healing assay further confirmed the inhibitory effect of L-*MBD2* siRNA on the TGF-β1-induced motility in A549 and B16F10 cells (Supplementary Fig. S7A, B).

To determine the impact of L-*MBD2* siRNA on the progression of EMT, A549 and B16F10 cells following TGF-β1 stimulation were immunostained with green fluorescence labeled antibodies against E-cadherin or Vimentin, and liposomes were labeled with Dil (red). It was noted that cells transfected with L-*MBD2* siRNA were coupled with enhanced E-cadherin (Fig. 4I, J) and attenuated Vimentin expression (Fig. 4K, L). Moreover, RT-PCR analysis of EMT markers (*CDH1*, CDH2 and *VIM*) in above cells obtained consistent results (Fig. 4M, N). Taken together, these results support that the liposome-based *MBD2* siRNA antagonizes TGF-β1-induced cancer cell migration, invasion coupled with attenuated EMT, and therefore, it could be a viable therapeutic approach against tumor metastasis.

### Administration of L-*MBD2* siRNA attenuates tumor metastasis

**Fig. 5:**
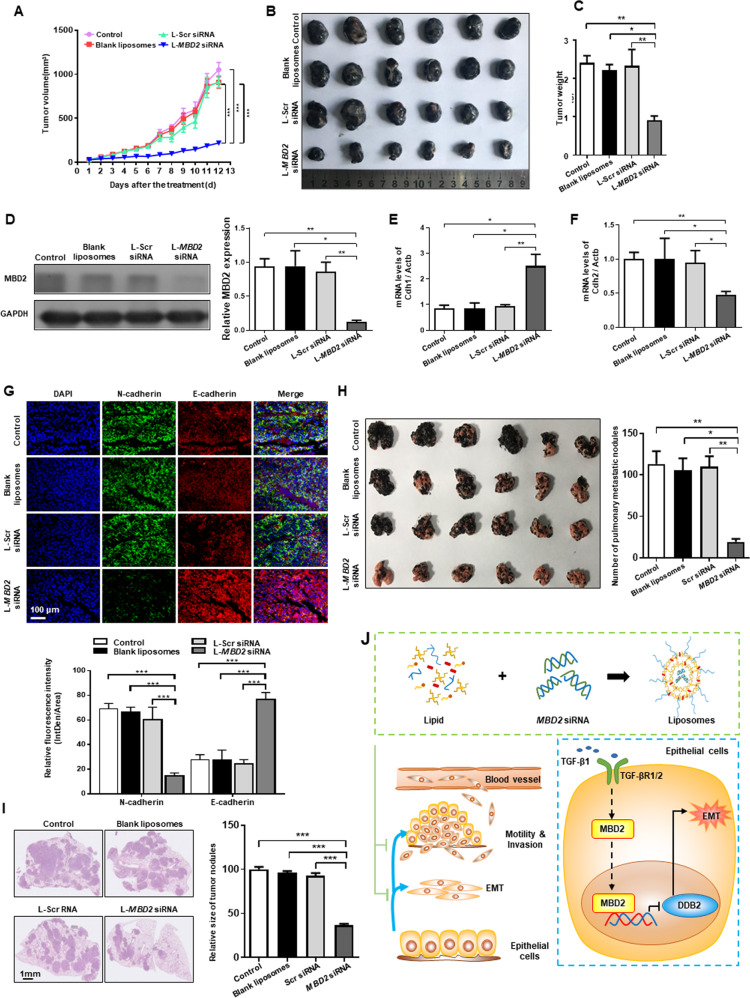
Administration of *MBD2* siRNA-loaded liposomes attenuated tumor metastasis in vivo. **A** The B16F10 melanoma tumor volume in each group was determined at the indicated time points. **B** Images of tumor nodules at the endpoint of the experiment. **C** The weight of the tumor nodules from each group at the endpoint of the experiment. **D** Western blot analysis of MBD2 in each group. **E**, **F** RT-PCR analysis of *Cdh1* (**E**) and *Cdh2* (**F**) in the indicated groups. **G** Representative images of E-cadherin and N-cadherin coimmunostaining in the tumor nodule sections. **H** Images of pulmonary metastatic nodules from the indicated groups. **I** Representative H&E staining of pulmonary metastatic nodules from the indicated groups. **J** MBD2 binds to methylated CpG DNA within the *DDB2* promoter and thus represses DDB2 expression, resulting in tumor metastasis. Intravenous administration of liposomes carrying *MBD2* siRNA suppressed tumor growth in an animal model bearing tumor metastasis. L-Scr siRNA: liposome-based Scrambled siRNA; L-*MBD2* siRNA: liposome-based *MBD2* siRNA. The data are presented as the mean ± SEM. *p < 0.05, **p < 0.01, ***p < 0.001.

Finally, we sought to assess the therapeutic potential of L-*MBD2* siRNA in tumor metastasis. The mice bearing subcutaneous B16F10 melanoma tumors with a volume of approximately 50 mm^3^ were intratumorally injected with PBS, empty liposomes, liposome-based scramble siRNA (L-Scrambled siRNA), and L-*MBD2* siRNA, respectively. Neither the empty liposomes nor the L-scramble siRNA exhibited perceptible impact on tumor growth inhibition (Fig. 5A, B). However, the L-*MBD2* siRNA treated mice were featured by a marked reduction in terms of tumor volume (Fig. 5A) or size (Fig. 5B) along with a decreased tumor mass (Fig. 5C). Likewise, no body mass difference was noted (Supplementary Fig. S8), demonstrating the outstanding biocompatibility of L-*MBD2* siRNA in vivo. Injection of L-*MBD2* siRNA significantly suppressed MBD2 expression in the tumors (Fig. 5D), which were accompanied by the robust increase of E-cadherin and reduction of N-cadherin expression, as determined by RT-PCR analysis (Fig. 5E, F) and immunostaining (Fig. 5G).

To further evaluate the anti-metastatic capability of L-*MBD2* siRNA, a metastasis model was established by intravenous injection of B16F10 melanoma cells. Pulmonary metastatic nodules were noted following 20 days of injection. Remarkably, L-*MBD2* siRNA strikingly suppressed the metastasis of B16F10 cells as compared to that in the other three groups, as manifested by the reduction for the number of tumor nodules in the lungs (Fig. 5H). Consistent with the above results, H&E staining also revealed alleviation of metastasis in the L-*MBD2* siRNA-treated group (Fig. 5I). Collectively, our data support the notion that L-*MBD2* siRNA could be a promising therapeutic approach against tumor metastasis and in situ tumor growth.

## Discussions

In the present study, we performed studies in human patients and animal models to elaborate the impact of MBD2, a reader for interpretation of DNA methylome-encoded information, on tumor metastasis. We demonstrated evidence suggesting an association between MBD2 expression and tumor metastasis in LUAD patients. Moreover, in vitro studies corroborated that MBD2 is involved in the regulation of TGF-β1-induced migration, invasion and EMT in multiple types of tumor cells. Mechanistically, MBD2 selectively binds to the methylated CpG DNA within the *DDB2* promoter, by which it represses DDB2 expression predisposing to tumor metastasis. Therefore, administration of liposomes carrying *MBD2 siRNA* suppressed tumor metastasis in the lung (Fig. 5J). Together, our data not only provide novel insights into the pathogenesis underlying tumor metastasis, but also demonstrate evidence supporting that *MBD2 siRNA* liposomes could be a viable approach against tumor metastasis in clinical settings.

Cancer is a class of diseases referring to a series of molecular changes to prefer uncontrolled cell growth and ultimately metastasize to other anatomical locations. Metastasis, a life-threatening event in cancer progression, contributes substantially to the current ranking of cancer as the second leading cause of death worldwide [28]. Although a great effort has recently been devoted to clarifying the pathoetiology underlying tumor metastasis, the exact molecular mechanisms, however, remain poorly understood. Aberrant DNA methylation has long been recognized in tumor development, and recently becomes more attractive due to its therapeutic potential [29]. Nevertheless, the severe side effect following global inhibition of DNA methylation limited the development of therapeutic strategies in clinical settings [30]. The DNA methylome-encoded information is read by a family of MBD proteins, through which they interpret the impact of DNA methylation on gene transcription while without affecting DNA methylation. Those properties rendered MBD proteins with feasibility to be therapeutic targets for tumor metastasis with acceptable side effects [31]. Particularly, MBD2 could be the most promising one, as deletion of MBD2 in mice does not generate any major deleterious effects [13, 32], supporting that MBD2 might be dispensable under physiological conditions.

Indeed, MBD2 has been shown to inhibit aberrantly methylated tumor suppressive genes by binding to the methylated CpG DNA within the promoter regions [33]. More recently, studies indicate that MBD2 probably modulates tumorigenesis in different types of cancer [34, 35]. In the past few years, our laboratory has concentrated on the effect of MBD2 on cancer progression. In Notch1-driven T-cell acute lymphoblastic leukemia (T-ALL), we first noted that MBD2 is required for the progression and maintenance of leukemia [13]. We showed evidence that MBD2 ablation under “steady-state” conditions not only causes a profound decrease in lymphocytes but also substantially impedes the progression of Notch1-driven T-ALL. In the current report, we focused on the role of MBD2 in pathogenesis of tumor metastasis, particularly in the metastasis of LUAD, as metastasis is one of the most important hallmarks of LUAD. Intriguingly, we demonstrated that higher expression of MBD2 was observed in tumors of LUAD patients with either lymphatic or distant tumor metastasis than in tumors in situ, but no significant difference in terms of MBD2 expression was noted between normal lung tissues and LUAD tumor tissues by analysis of paired samples from 73 LUAD patients (Fig. 1) and the data from 58 pairs of LUAD patients from the Oncomine database (Supplementary Fig. 1). These observations strongly suggest that MBD2 might be a critical prognostic marker for tumor metastasis. Indeed, the data from the Human Protein Atlas illustrated that higher MBD2 levels were correlated with poorer outcome in patients with LUAD.

EMT has been reported to increase the motility and invasiveness of cancer cells by repressing the expression of epithelial markers and promoting the expression of mesenchymal markers, which in turn facilitates the formation of metastases [6]. Abundant evidence suggests that TGF-β1 can promote the migration and invasion of various cancer cells [7, 36]. We thus stimulated A549, H1975 and B16F10 cells with TGF-β1 to assess the impact of MBD2 on cancer cell EMT process. We demonstrated convincing evidence that knockdown of MBD2 markedly inhibited TGF-β1-induced cell migration and invasion coupled with attenuated EMT. However, in contrast to our observation, Pei and colleagues reported that lower MBD2 expression was significantly correlated with higher tumor stage and metastasis *via* deteriorating the EMT process [37]. This discrepancy is most likely caused by the differences of experimental approaches employed. Specifically, they assessed the EMT process in A549 cells without TGF-β1 induction, while we stimulated the cancer cells with TGF-β1, as TGF-β1 is highly enriched in the tumor microenvironment and essential for tumor metastasis. Nevertheless, regardless of which point of view is more persuasive, all of these findings suggest the implication of MBD2 in modulating tumor metastasis, thereby contributing to the pathogenesis of cancer progression.

Extensive studies support the therapeutic potential for cancer metastasis by inhibiting EMT program, which highlights an urgent need for developing effective anticancer drugs to target the TGF-β1 signaling. Generally, TGF-β1 activates the ALK5 type I receptor (which phosphorylates SMAD2/3) as well as noncanonical (e.g., Src kinase, EGFR, JAK/STAT, P53) pathways that jointly drive cancer progression [38, 39]. In nucleotide excision repair, P53 functions as a transcription factor to regulate both the basal and DNA damage-inducible expression of a set of genes involved in DNA repair, including DDB2 [40], which has been proposed to act as a suppressor of tumorigenesis by transcriptionally repressing the expression of genes essential for EMT program, such as Snail and Zeb1 [41]. Indeed, our RNA-seq data revealed that TGF-β1-treated A549 cells transfected with *MBD2 siRNA* were characterized by a gain of the DDB2 signature, which was further confirmed by RT-PCR. In fact, TGF-β1 induced A549 cells to undergo a DNA hypermethylation within the *DDB2* promoter along with the induction of MBD2 overexpression, which in turn bound to the methylated CpG DNA within the *DDB2* promoter to suppress its expression, thereby activating and sustaining the EMT program.

Over the past few decades, remarkable scientific progress has been made in cancer treatments, such as surgical therapy, chemotherapy and radiotherapy. Improvements in the use of these therapies have improved the disease-free survival of cancer patients [42]. However, there are still many challenges in cancer treatment unsettled. Regarding the discovery of various proto-oncogenes and tumor suppressor genes, gene therapy has become a popular research direction in the treatment of metastatic cancers [43]. Nanovehicles have been extensively applied for the effective delivery of siRNA or chemotherapeutics [44]. In particular, positively charged cationic liposomes have been widely used as siRNA and chemotherapeutic carriers due to their high loading capacity and significantly enhanced cellular internalization through adsorptive interactions with the tumor cell membrane [45]. For example, patisiran (Onpattro) has been approved by the Food and Drug Administration (FDA) in 2018 as the first lipid nanoparticle-based siRNA drug for the treatment of hereditary transthyretin-mediated amyloidosis, which translates such an idea into the clinical practice [46]. The most exciting discovery in this study was that administration of *MBD2* siRNA-loaded liposomes significantly attenuated xenograft metastatic potential in animal models, highlighting a promising prospect and important application value of *MBD2* siRNA-loaded liposomes in clinical settings. However, our study still has some limitations. First, the cohort of patients with metastatic LUAD was relatively small. More patients are needed to verify the association of MBD2 and tumor metastasis. Second, MBD2 was upregulated in A549, H1975 and B16F10 cells after TGF-β1 stimulation. However, the mechanism underlying MBD2 overexpression is far from clear. In our recent study, TGF-β1 could upregulate the expression of MBD2 via the canonical SMAD pathway in fibroblasts [47]. We speculated that the expression of MBD2 induced by TGF-β1 might be depended on the canonical SMAD pathway in tumor cells as well, but it needs to be confirmed. Third, the expression of MBD2 may facilitate tumor growth, as evidenced by the significantly lower tumor volume, size and mass in the L-*MBD2* siRNA treated mice. We believe that this phenotype caused by the enhancing effect of *MBD2* siRNA on inducing cancer cell apoptosis (Supplementary Fig. 8H). However, the detailed mechanism needs to be further explored.

In summary, this study indicated that MBD2 facilitates cancer metastasis, and therefore, knockdown of MBD2 inhibited TGF-β1-induced EMT, migration and invasion of cancer cells. Mechanistically, MBD2 selectively binds to the methylated CpG DNA within the *DDB2* promoter region, by which MBD2 suppresses DDB2 expression to motivate the EMT program. Given that MBD2 itself does not influence DNA methylation and seems to be dispensable for the biological processes under physiological conditions [32], MBD2 is likely to be a viable target to suppress cancer metastasis. Indeed, intratracheal instillation of liposomes carrying *MBD2* siRNA substantially inhibited tumor growth and metastasis in a B16F10 tumor-bearing animal model. Together, our data provide important proof of principle for MBD2 as a useful metastasis biomarker and describe liposome-based *MBD2* siRNA as a candidate anticancer agent in clinical settings.

## Supplementary information

supplementary figure

supplementary table

western blot

Reproducibility checklist

## Data Availability

The data used in the current study are all available from the corresponding author upon reasonable request.

## Materials availability

The materials used in the current study are all available from the corresponding author upon reasonable request.
