# Supplementary material for: MBD2 facilitates tumor metastasis by mitigating DDB2 expression
Source: Cell Death Dis. 2023 May 4;14(5):303. doi: 10.1038/s41419-023-05804-1 (PMC10160113; doi:10.1038/s41419-023-05804-1)
Supplement: Supplementary file 1 — supplementary figure [file 41419_2023_5804_MOESM1_ESM.docx]

**MBD2 facilitates tumor metastasis by mitigating DDB2 expression**

**Running title:** The role of MBD2 in tumor metastasis

Lei Zhang^1, #^, Siyuan Wang^2, #^, Guo-Rao Wu^1^, Huihui Yue^1^, Ruihan Dong^1^, Shu Zhang^1^, Qilin Yu^1^, Ping Yang^1^, Jianping Zhao^1^, Huilan Zhang^1^, Jun Yu^3^, Xianglin Yuan^4^, Weining Xiong^5^, Xiangliang Yang^6, *^, Tuying Yong^6, *^, Cong-Yi Wang^1, *^

^1^ Department of Respiratory and Critical Care Medicine, The Center for Biomedical Research, NHC Key Laboratory of Respiratory Diseases, Tongji Hospital, Tongji Medical College, Huazhong University of Sciences and Technology, 1095 Jiefang Ave, Wuhan 430030, China.

^2^ Department of Gerontology, The Central Hospital of Wuhan, Tongji Medical College, Huazhong University of Science and Technology, Wuhan, China.

^3^ Department of Thoracic Surgery, Tongji Hospital, Tongji Medical College, Huazhong University of Sciences and Technology, 1095 Jiefang Ave, Wuhan 430030, China.

^4^ Department of Oncology, Tongji Hospital, Tongji Medical College, Huazhong University of Science and Technology, Wuhan 430030, China.

^5^ Department of Respiratory and Critical Care Medicine, Shanghai Key Laboratory of Tissue Engineering, Shanghai Ninth People's Hospital, Shanghai Jiaotong University School of Medicine, 639 Zhizaoju Lu, Shanghai, 200011, China.

^6^ National Engineering Research Center for Nanomedicine, College of Life Science and Technology, Huazhong University of Science and Technology, Wuhan, 430074, China.

^#^These authors contributed equally to this work.

*Correspondence: Cong-Yi Wang (Tel: 86-27-6937-8458; E-mail: [wangcy@tjh.tjmu.edu.cn](mailto:wangcy@tjh.tjmu.edu.cn)), the Center for Biomedical Research, Tongji Hospital Research Building, Tongji Hospital, Tongji Medical College, Huazhong University of Science and Technology, Wuhan, China; or Tuying Yong (Tel: 86-27-8779-2147; E-mail: yongty2018@hust.edu.cn) or Xiangliang Yang (Tel: 86-27-8779-2147; E-mail: [yangxl@mail.hust.edu.cn](mailto:yangxl@mail.hust.edu.cn)), National Engineering Research Center for Nanomedicine, College of Life Science and Technology, Huazhong University of Science and Technology, Wuhan, China.

**Supplementary Figures and Legends**

**
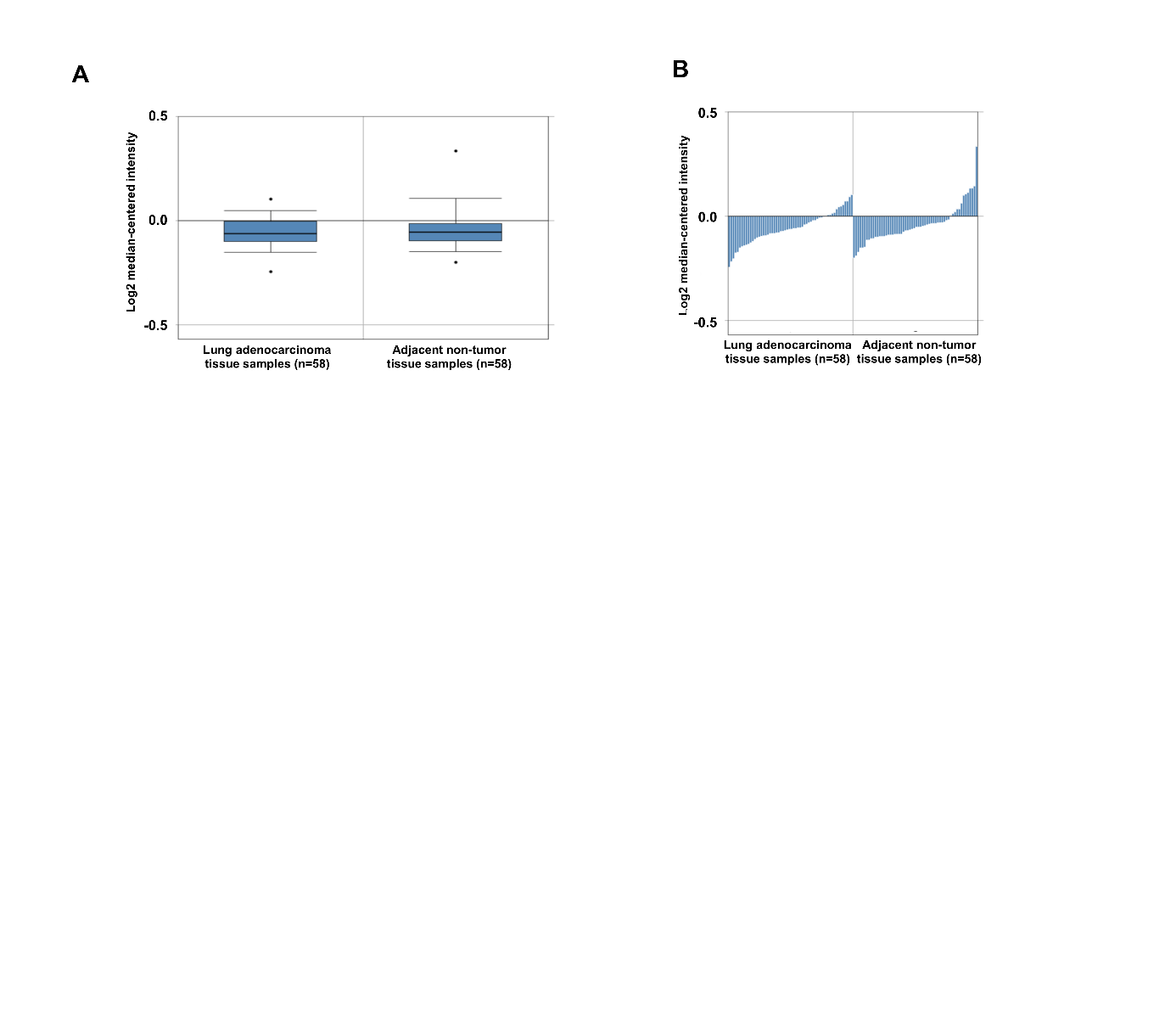
**

**Supplementary Fig. 1** **A-B** Analysis of *MBD2* gene expression in 58 pairs of lung adenocarcinoma tissue samples and adjacent nontumor tissue samples using the Oncomine database.


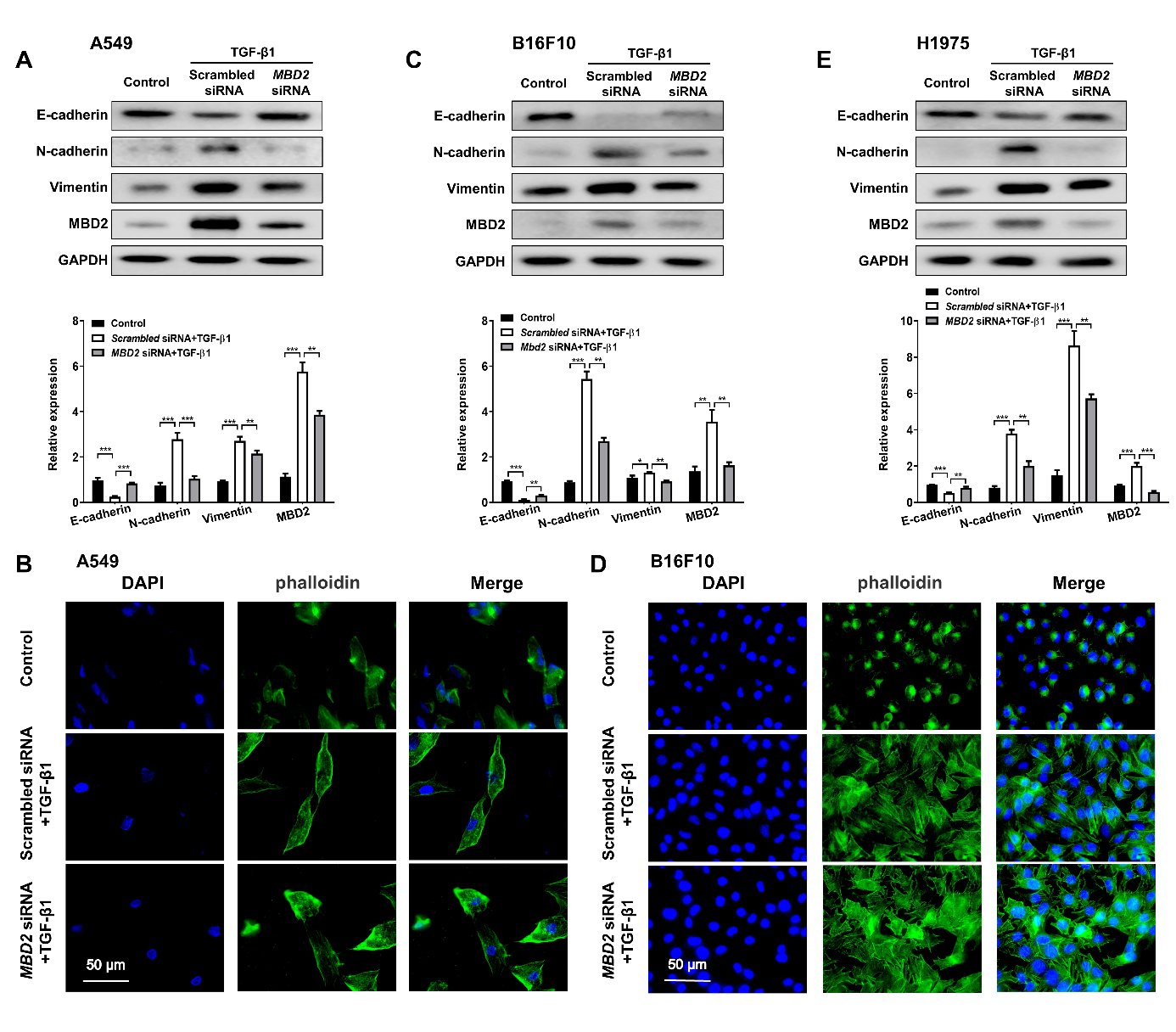


**Supplementary Fig. 2** Western blot analysis of E-cadherin, N-cadherin, Vimentin and MBD2 in A549 **(A)**, B16F10 cells **(C)** and NCI-H1975 cells **(E)** following *MBD2* siRNA transfected. The morphology of TGF-β1 treated A549 (**B**) and B16F10 cells (**D**) after *MBD2* siRNA transfected. All images were acquired at 400× magnification. The data are presented as the mean ± SEM. **p* < 0.05, ***p* < 0.01, ****p* < 0.001.

**
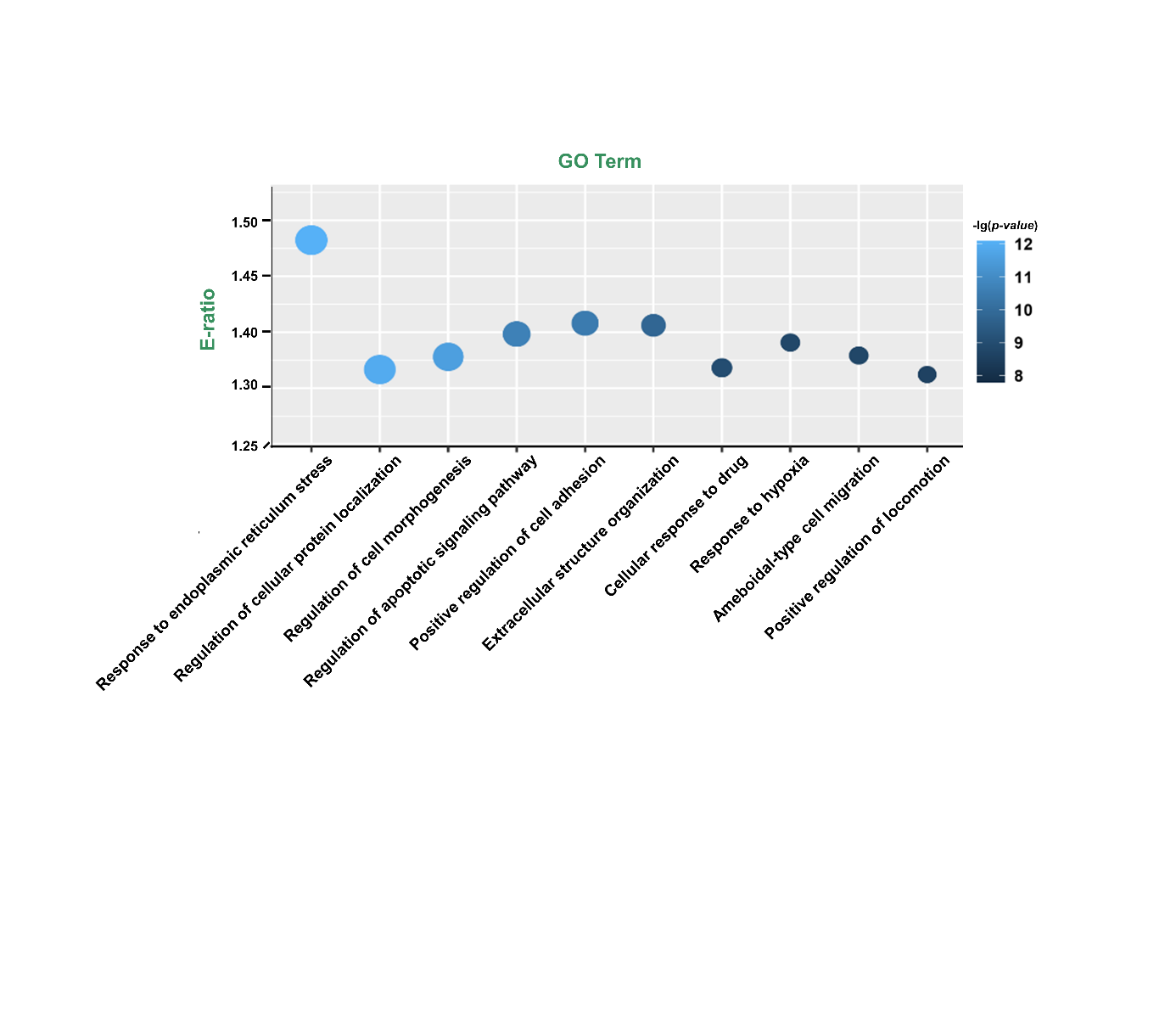
**

**Supplementary Fig. 3** GO enrichment analysis of upregulated DEGs with a fold change > 1.5.


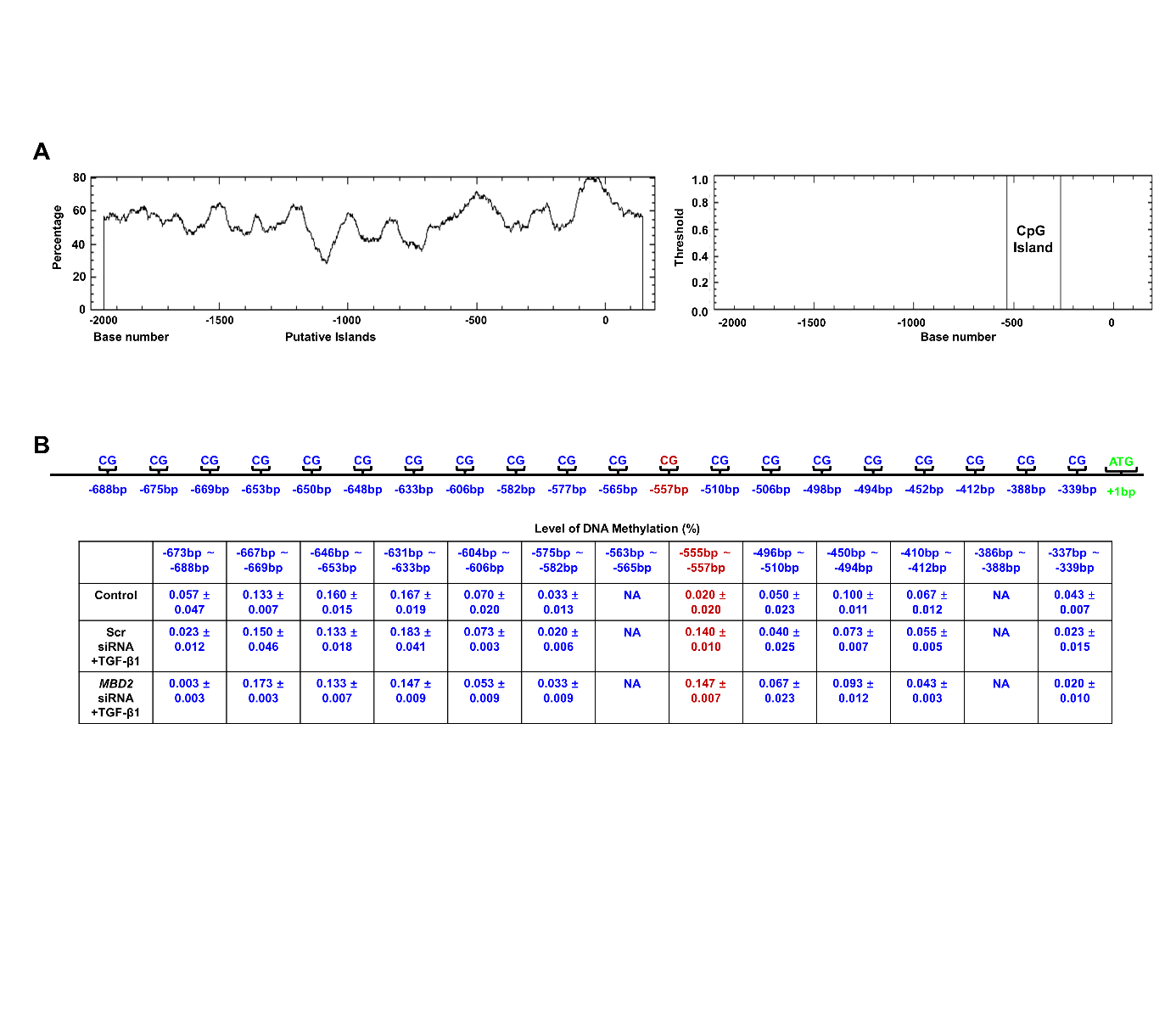


**Supplementary Fig. 4 A** Predicted CpG islands in the *DDB2* promoter. **B** A diagram showing the interacted regions in *DDB2* promoter. Methylated CpG DNA in the region (from -555bp to -557bp, the transcription start site as +1) was highlighted within the *DDB2* promoter.

**
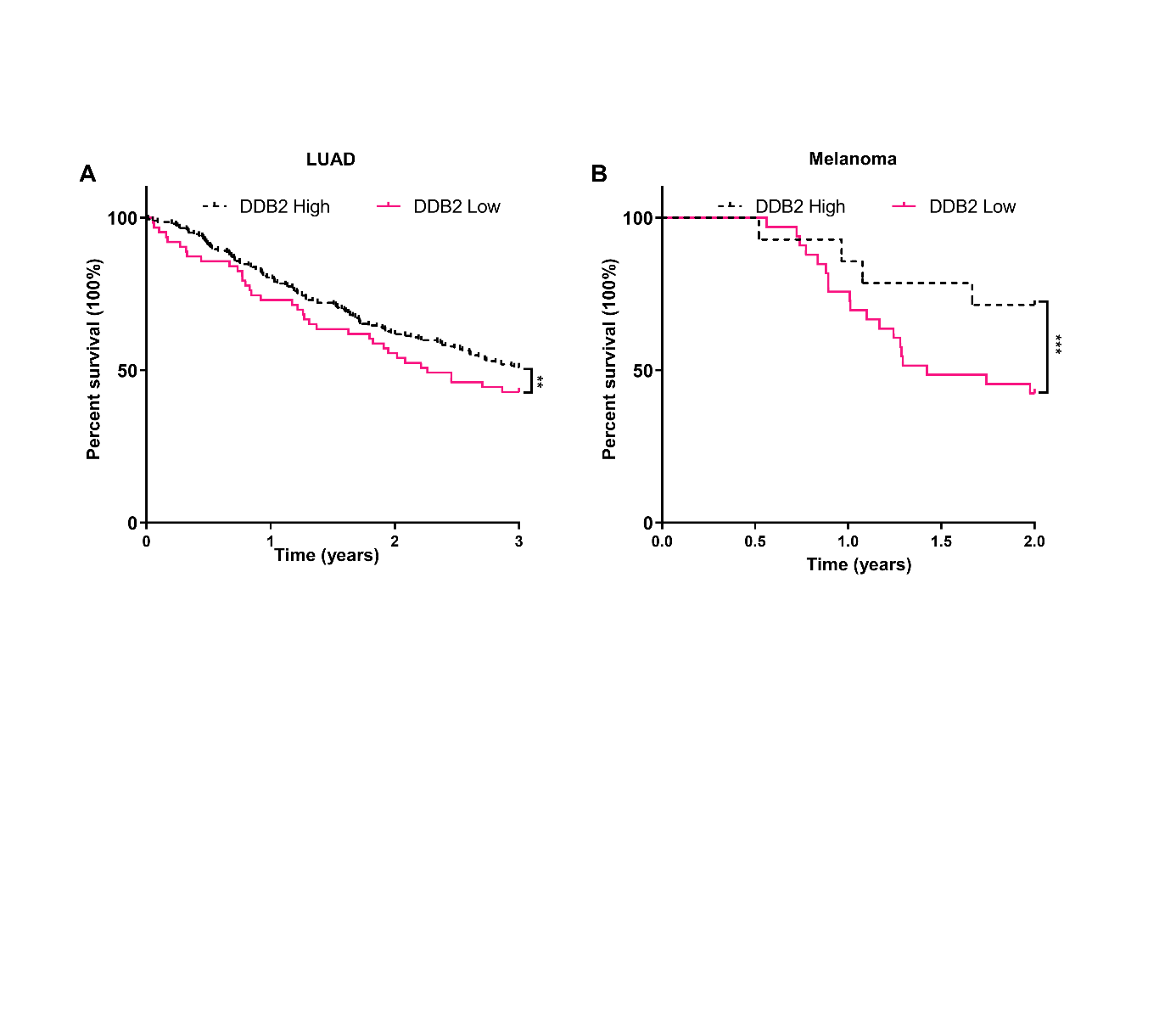
**

**Supplementary Fig. 5** Prognostic value of DDB2 expression in patients with LUAD **(A)** or melanoma **(B)**. Data derived from the Human Protein Atlas database. ***p* < 0.01, ****p* < 0.001.

**
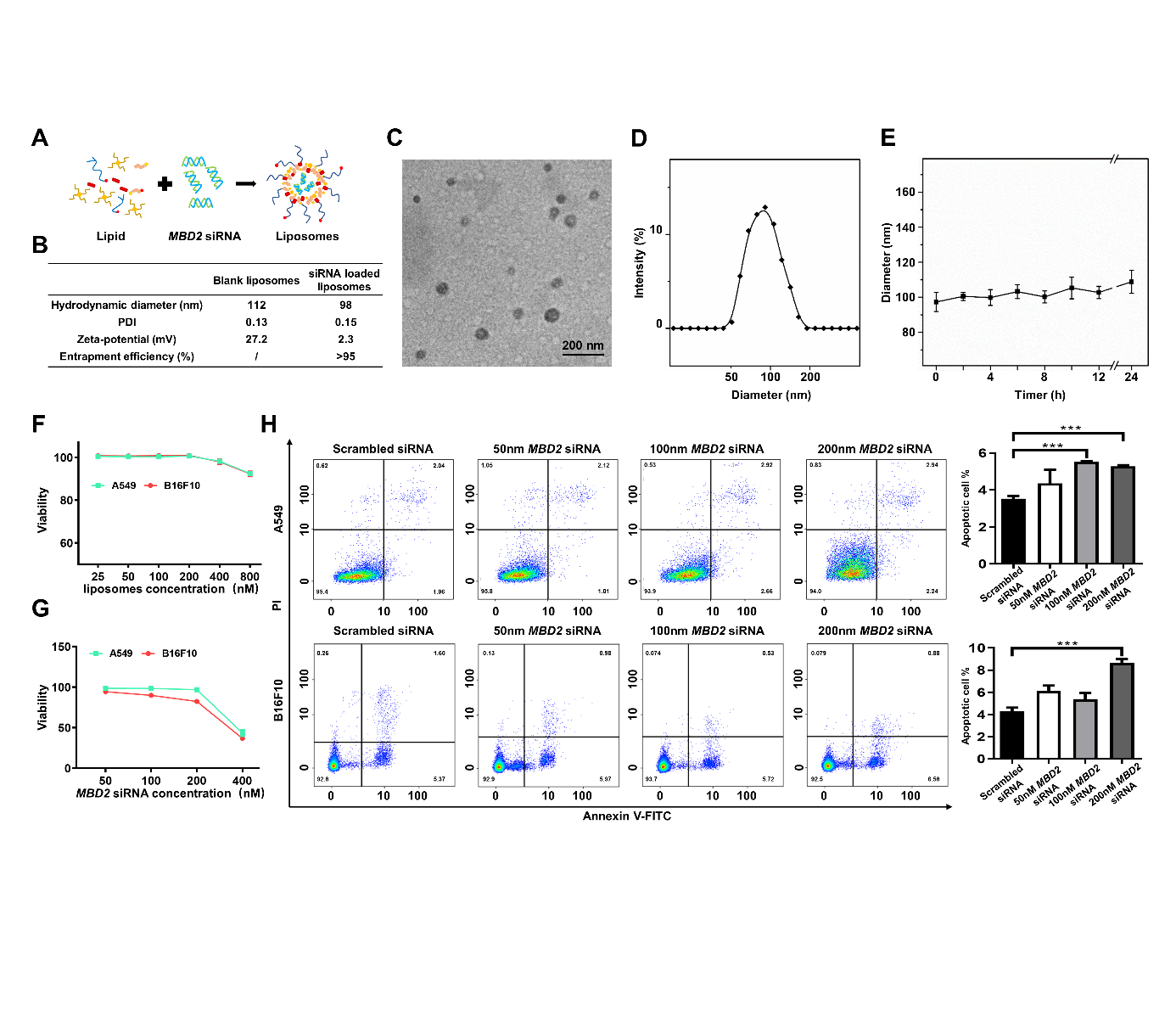
**

**Supplementary Fig. 6** **A** Preparation of *MBD2* siRNA-loaded liposomes. **B** The hydrodynamic diameter, PDI, zeta potential and entrapment efficiency of liposomes (empty or siRNA-loaded). **C** Representative TEM image of siRNA-loaded liposomes. **D** Hydrodynamic diameter distribution of siRNA-loaded liposomes. **E** Colloidal stability of siRNA-loaded liposomes in PBS. CCK-8 assay **(F and G)** and apoptosis assay **(H)** of cells transfected with different concentrations of *MBD2* siRNA-loaded liposomes for 48 h. The data are presented as the mean ± SEM. ****p* < 0.001.

**
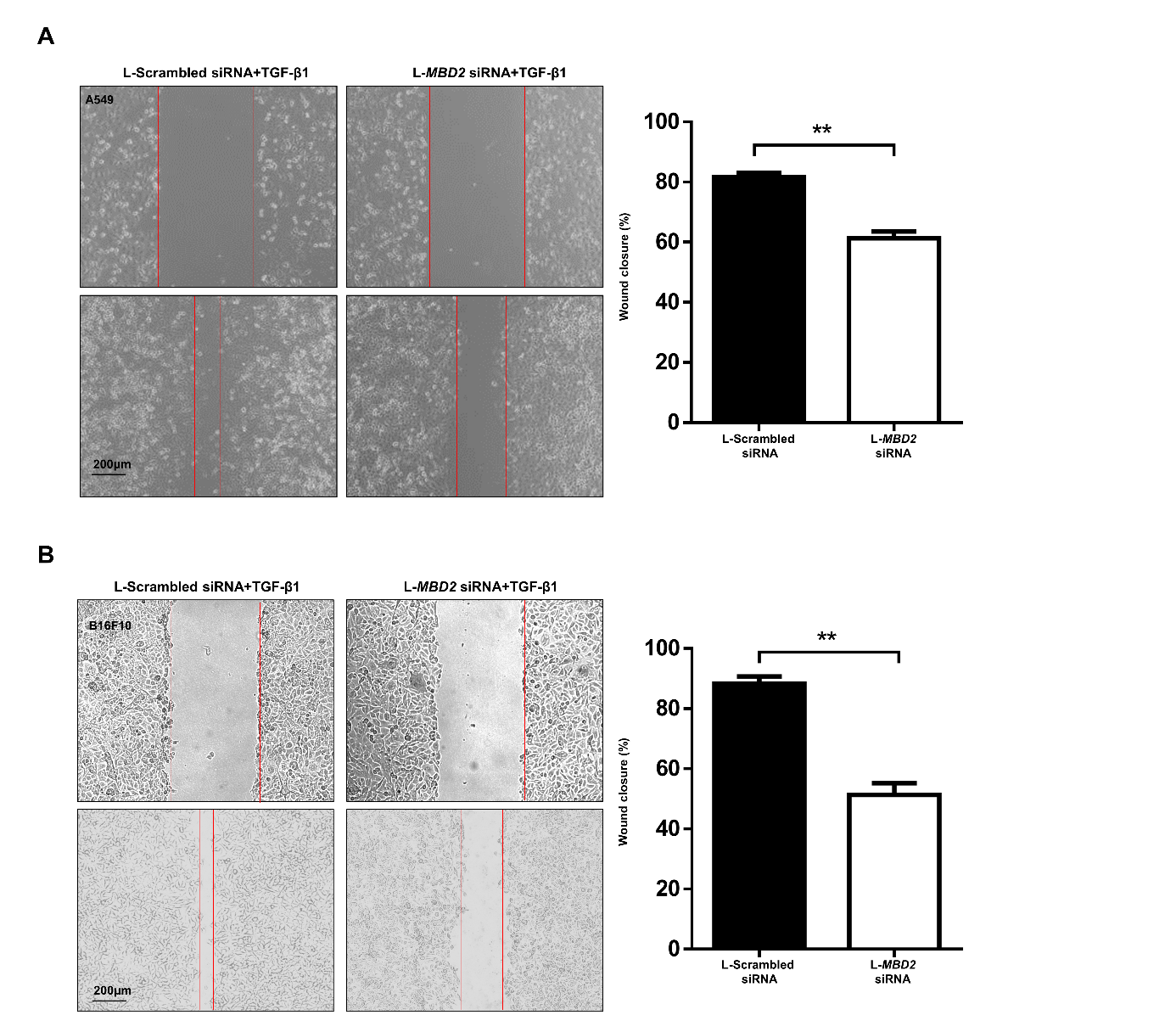
**

**Supplementary Fig. 7** A wound healing assay of TGF-β1-induced A549 **(A)** and B16F10 **(B)** cells after transfected with *MBD2* siRNA-loaded liposomes. The data are presented as the mean ± SEM. ***p* < 0.01.


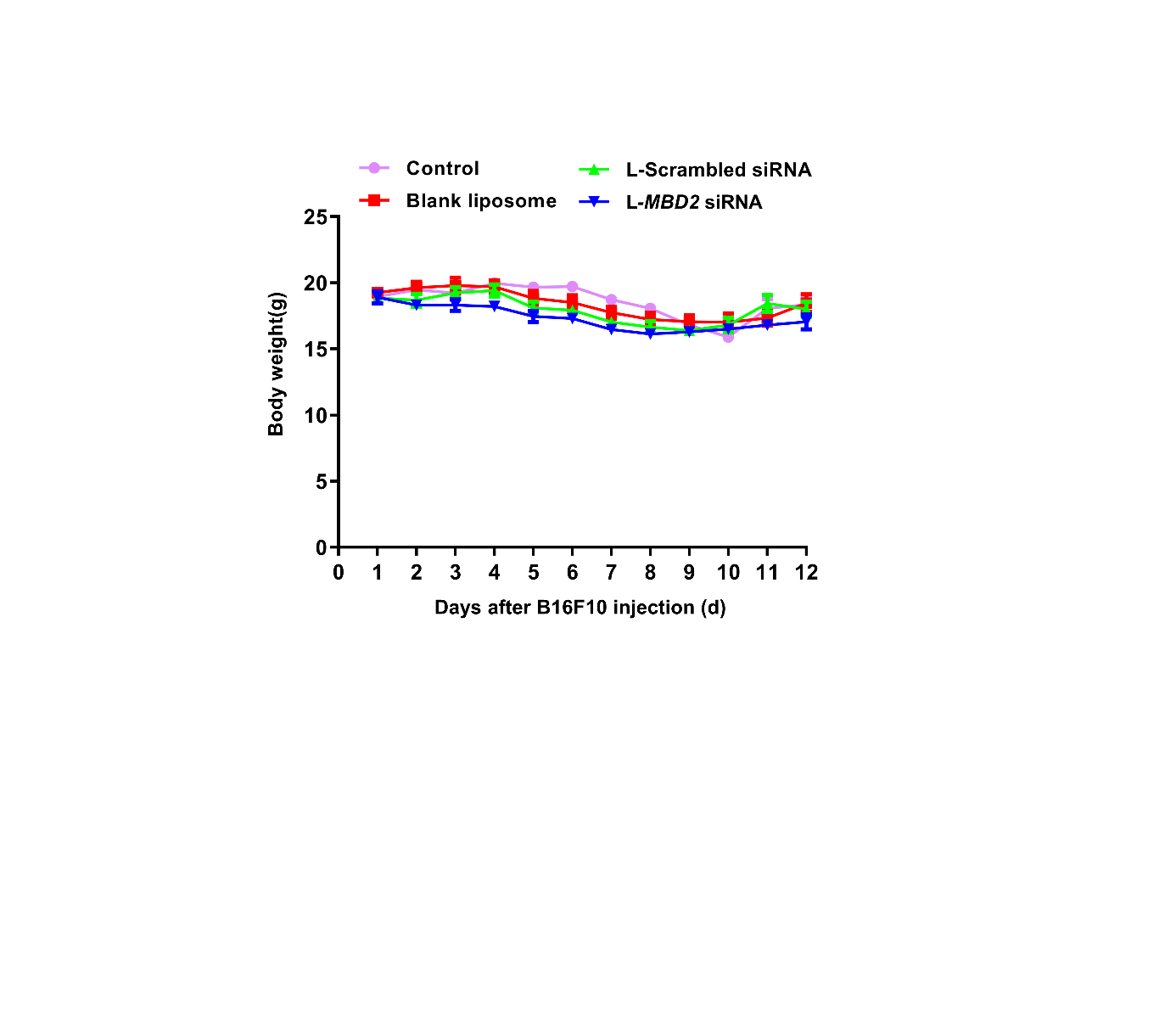


**Supplementary Fig. 8** The weights of mice were determined at different time points after subcutaneous injection of B16F10 cells.
