# Supplementary material for: MBD2 facilitates tumor metastasis by mitigating DDB2 expression
Source: Cell Death Dis. 2023 May 4;14(5):303. doi: 10.1038/s41419-023-05804-1 (PMC10160113; doi:10.1038/s41419-023-05804-1)
Supplement: Supplementary file 2 — supplementary table [file 41419_2023_5804_MOESM2_ESM.docx]

**MBD2 facilitates tumor metastasis by mitigating DDB2 expression**

**Running title:** The role of MBD2 in tumor metastasis

Lei Zhang^1, #^, Siyuan Wang^2, #^, Guo-Rao Wu^1^, Huihui Yue^1^, Ruihan Dong^1^, Shu Zhang^1^, Qilin Yu^1^, Ping Yang^1^, Jianping Zhao^1^, Huilan Zhang^1^, Jun Yu^3^, Xianglin Yuan^4^, Weining Xiong^5^, Xiangliang Yang^6, *^, Tuying Yong^6, *^, Cong-Yi Wang^1, *^

^1^ Department of Respiratory and Critical Care Medicine, The Center for Biomedical Research, NHC Key Laboratory of Respiratory Diseases, Tongji Hospital, Tongji Medical College, Huazhong University of Sciences and Technology, 1095 Jiefang Ave, Wuhan 430030, China.

^2^ Department of Gerontology, The Central Hospital of Wuhan, Tongji Medical College, Huazhong University of Science and Technology, Wuhan, China.

^3^ Department of Thoracic Surgery, Tongji Hospital, Tongji Medical College, Huazhong University of Sciences and Technology, 1095 Jiefang Ave, Wuhan 430030, China.

^4^ Department of Oncology, Tongji Hospital, Tongji Medical College, Huazhong University of Science and Technology, Wuhan 430030, China.

^5^ Department of Respiratory and Critical Care Medicine, Shanghai Key Laboratory of Tissue Engineering, Shanghai Ninth People's Hospital, Shanghai Jiaotong University School of Medicine, 639 Zhizaoju Lu, Shanghai, 200011, China.

^6^ National Engineering Research Center for Nanomedicine, College of Life Science and Technology, Huazhong University of Science and Technology, Wuhan, 430074, China.

^#^These authors contributed equally to this work.

*Correspondence: Cong-Yi Wang (Tel: 86-27-6937-8458; E-mail: [wangcy@tjh.tjmu.edu.cn](mailto:wangcy@tjh.tjmu.edu.cn)), the Center for Biomedical Research, Tongji Hospital Research Building, Tongji Hospital, Tongji Medical College, Huazhong University of Science and Technology, Wuhan, China; or Tuying Yong (Tel: 86-27-8779-2147; E-mail: yongty2018@hust.edu.cn) or Xiangliang Yang (Tel: 86-27-8779-2147; E-mail: [yangxl@mail.hust.edu.cn](mailto:yangxl@mail.hust.edu.cn)), National Engineering Research Center for Nanomedicine, College of Life Science and Technology, Huazhong University of Science and Technology, Wuhan, China.

**Supplementary Table 1. Characteristics of the lung adenocarcinoma patients**

|  | Lung adenocarcinoma samples |
| --- | --- |
| Variable |  |
| Age^1^, years |  |
| ≤58 | 33 |
| >58 | 36 |
| Gender |  |
| Male | 31 |
| Female | 38 |
| Smoking |  |
| Never | 46 |
| Current or past smoker | 23 |
| Differentiation |  |
| Well | 22 |
| Moderately | 23 |
| Poorly | 24 |
| Stage |  |
| IA, IB | 7 |
| IC | 19 |
| IIA | 28 |
| IIB | 7 |
| III, IV | 8 |
| N staging |  |
| 0 | 39 |
| 1 | 11 |
| 2-3 | 19 |
| M staging |  |
| 0 | 64 |
| 1 | 5 |

N, node; M, metastasis.

^1^The mean age at diagnosis is 58.8 years in lung adenocarcinoma patients. Samples are divided into two groups according to the mean age.

**Supplementary Table 2. The primers used for RT-PCR**

| Gene name | F-primer (5’-3’) | R-primer (5’-3’) |
| --- | --- | --- |
| *MBD2 (Homo)* | AAG TGA TCC GAA AAT CTG GGC | TGC CAA CTG AGG CTT GCT TC |
| *CDH1 (Homo)* | CGA GAG CTA CAC GTT CAC GG | GGG TGT CGA GGG AAA AAT AGG |
| *Cdh1 (Mus)* | CAG TTC CGA GGT CTA CAC CTT | TGA ATC GGG AGT CTT CCG AAA A |
| *CDH2 (Homo)* | TCA GGC GTC TGT AGA GGC TT | ATG CAC ATC CTT CGA TAA GAC TG |
| *Cdh2 (Mus)* | AGG CTT CTG GTG AAA TTG CAT | GTC CAC CTT GAA ATC TGC TGG |
| *VIM (Homo)* | GAC GCC ATC AAC ACC GAG TT | CTT TGT CGT TGG TTA GCT GGT |
| *Vim (Mus)* | CGT CCA CAC GCA CCT ACA G | GGG GGA TGA GGA ATA GAG GCT |
| *TP53 (Homo)* | CAG CAC ATG ACG GAG GTT GT | TCA TCC AAA TAC TCC ACA CGC |
| *DDB2 (Homo)* | ACC TCC GAG ATT GTA TTA CGC C | TCA CAT CTT CTG CTA GGA CCG |
| *GAPDH Homo)* | GCG ACA CCC ACT CCT CCA CCT TT | TGC TGT AGC CAA ATT CGT TGT CAT A |
| *Gapdh (Mus)* | AGG TCG GTG TGA ACG GAT TTG | GGG GTC GTT GAT GGC AAC A |
