# Supplementary material for: MBD2 facilitates tumor metastasis by mitigating DDB2 expression
Source: Cell Death Dis. 2023 May 4;14(5):303. doi: 10.1038/s41419-023-05804-1 (PMC10160113; doi:10.1038/s41419-023-05804-1)
Supplement: Supplementary file 3 — western blot [file 41419_2023_5804_MOESM3_ESM.docx]

Western blot：

Figure 2A:


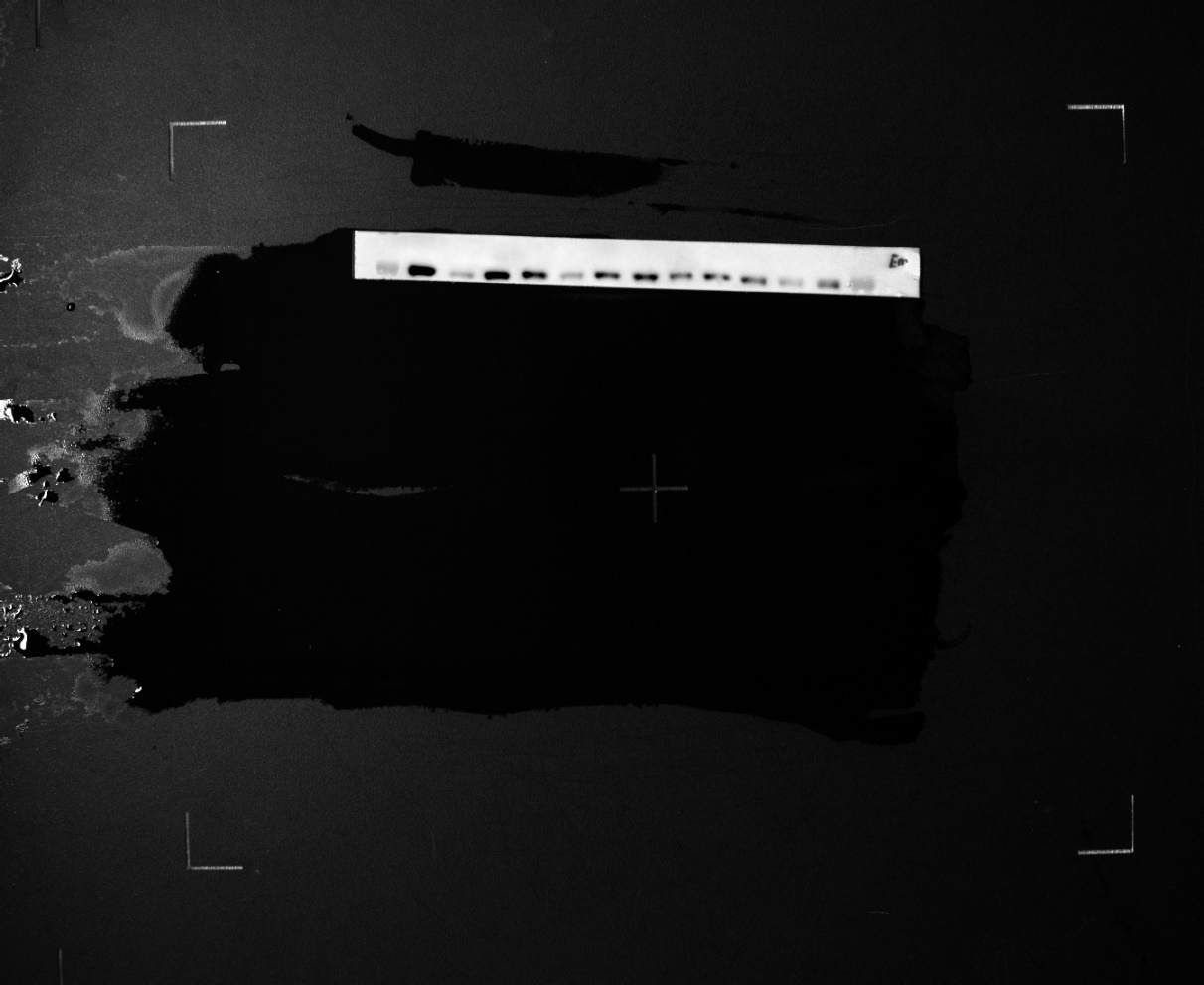

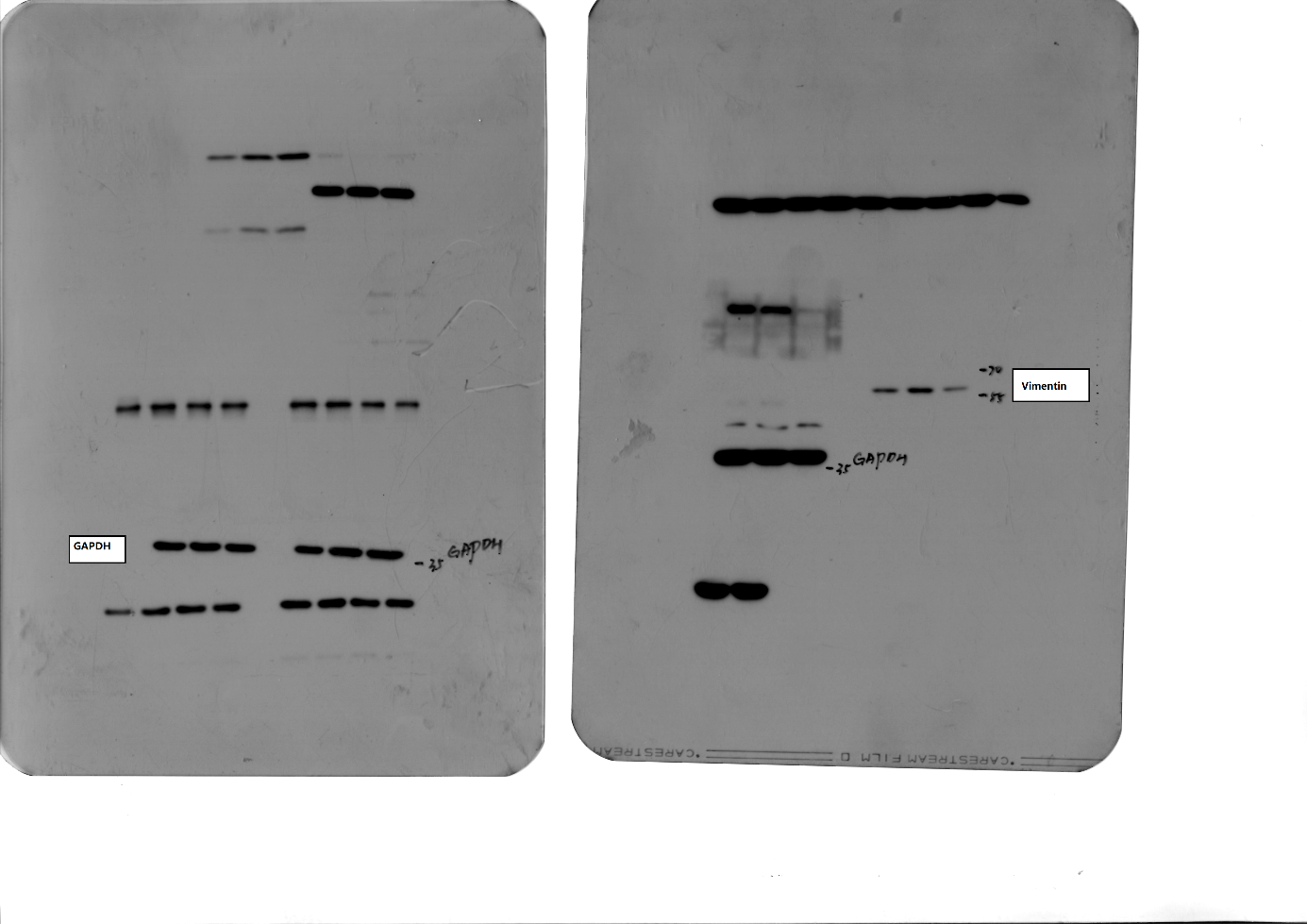

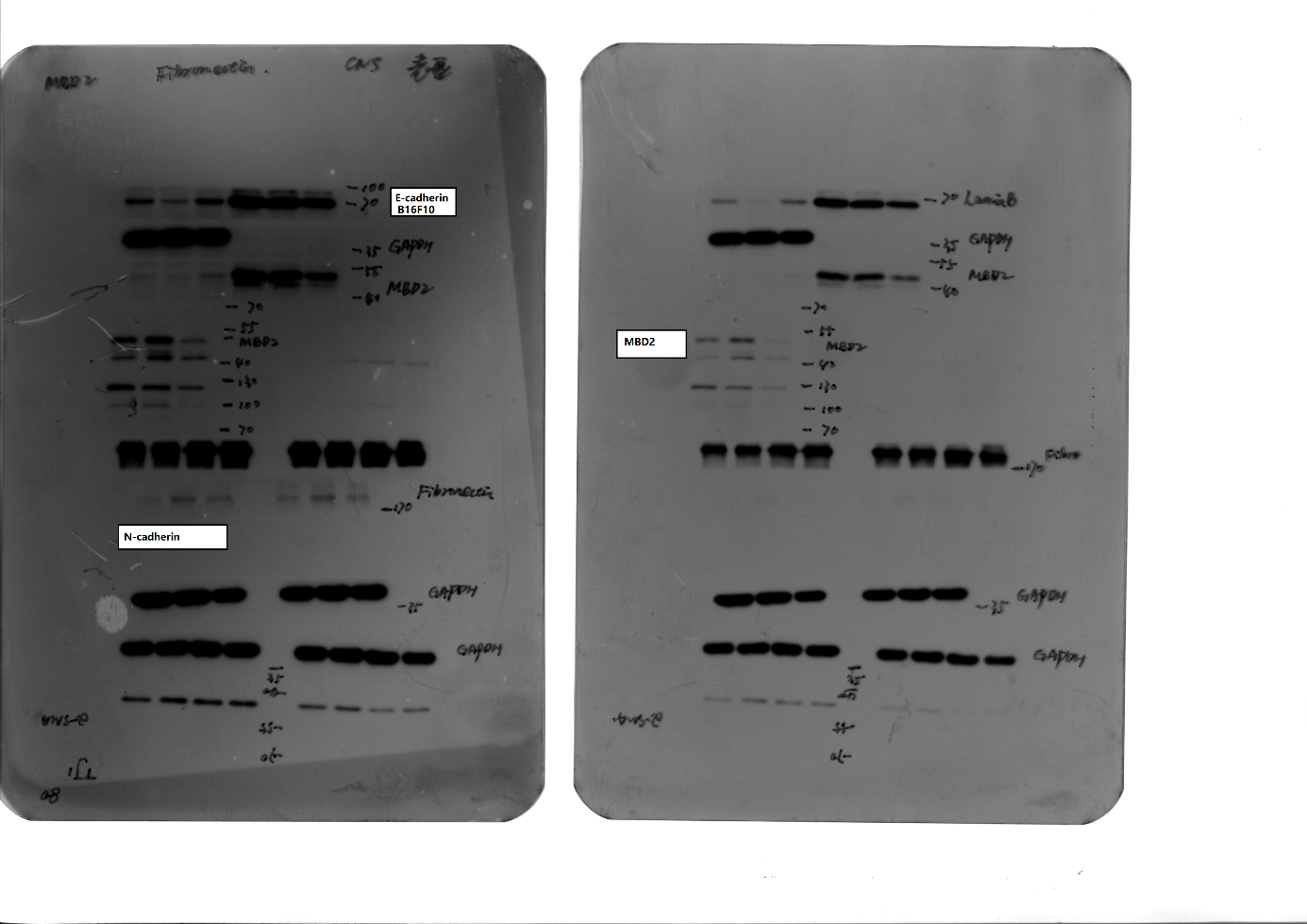


A549 N-cadherin

A549 MBD2

A549 E-cadherin

A549 Vimentin

A549 GAPDH

Figure 2C:


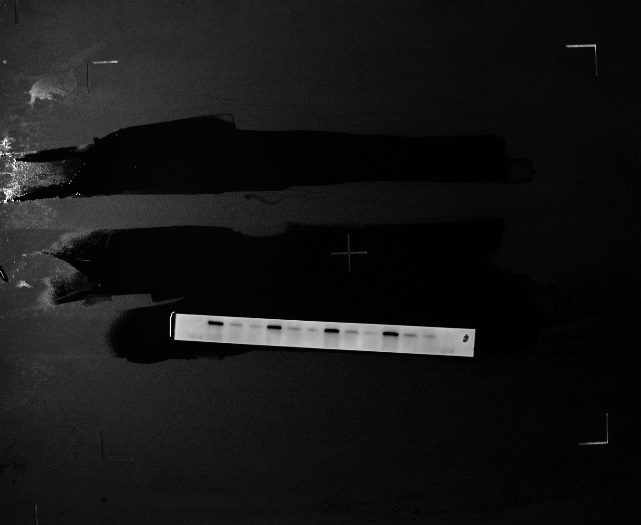

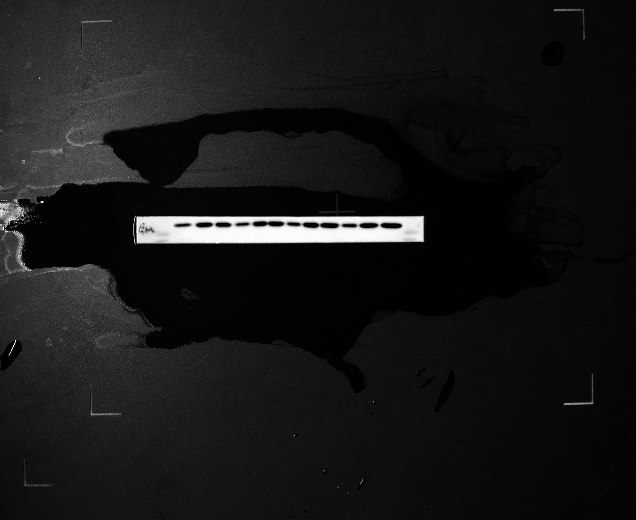

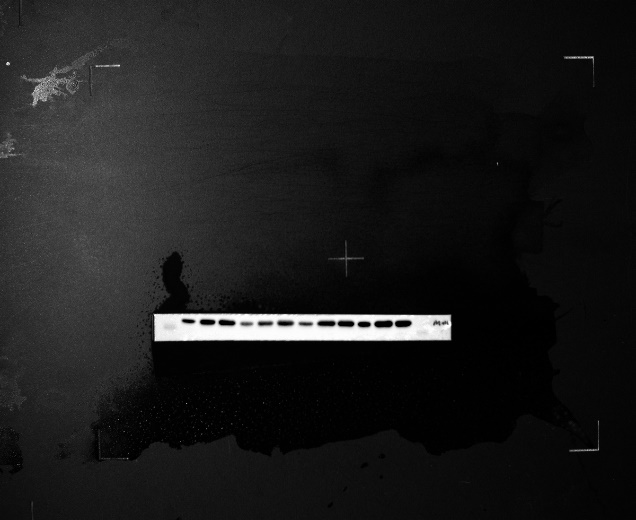

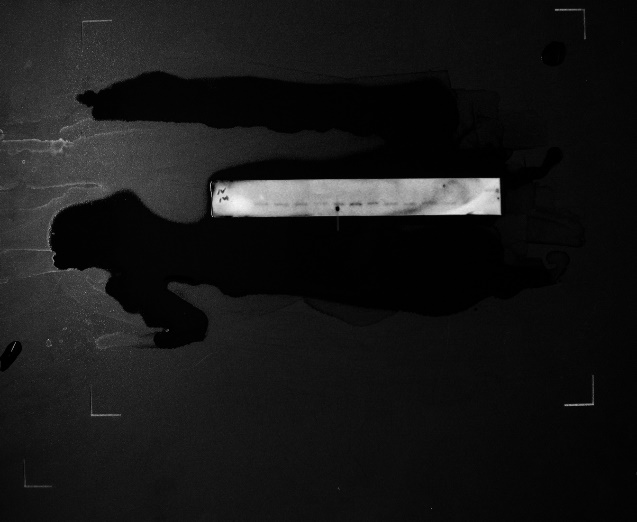


A549 N-cadherin

A549 MBD2

A549 GAPDH

A549 E-cadherin

Figure 2D：(B16F10 cells)


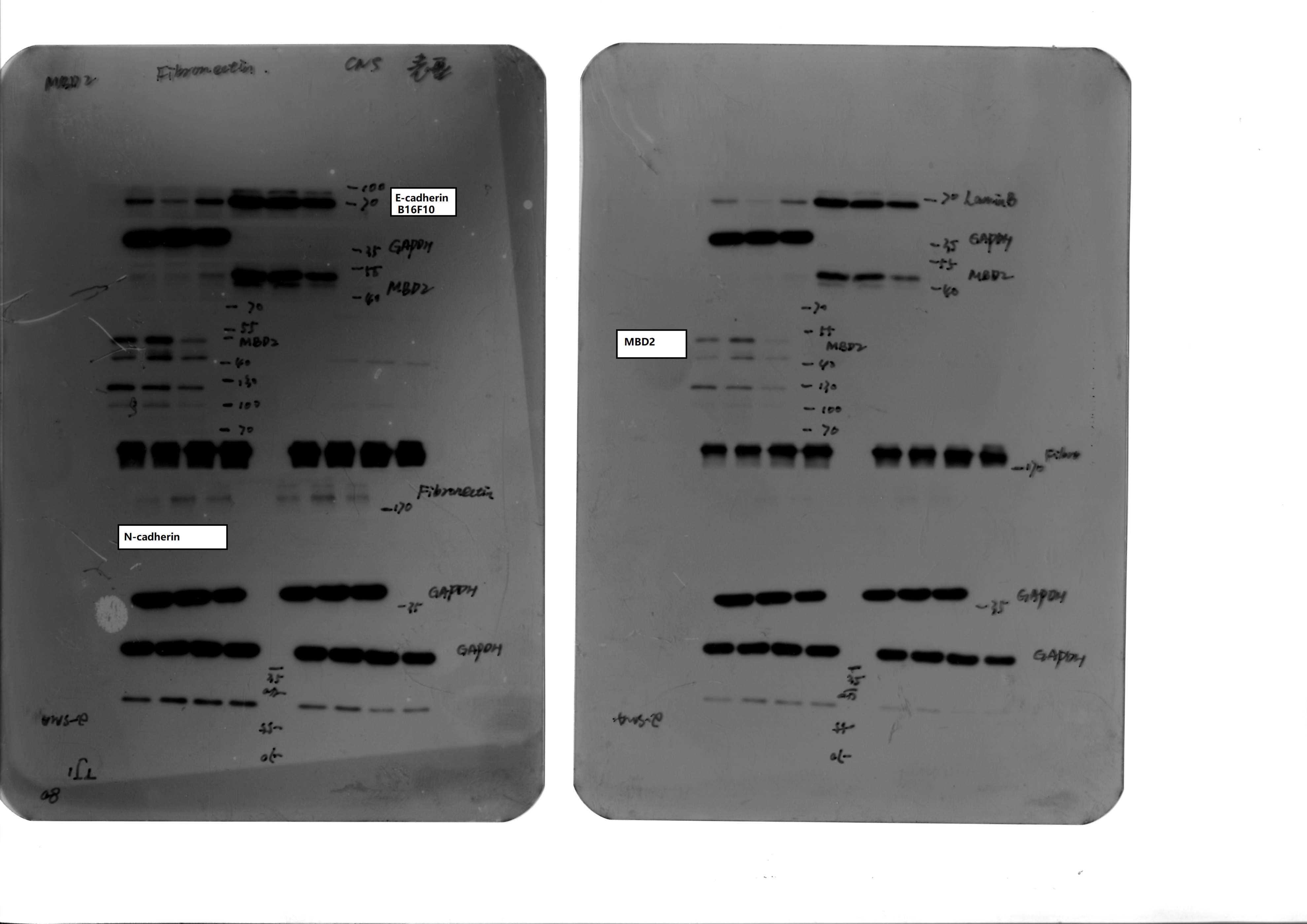

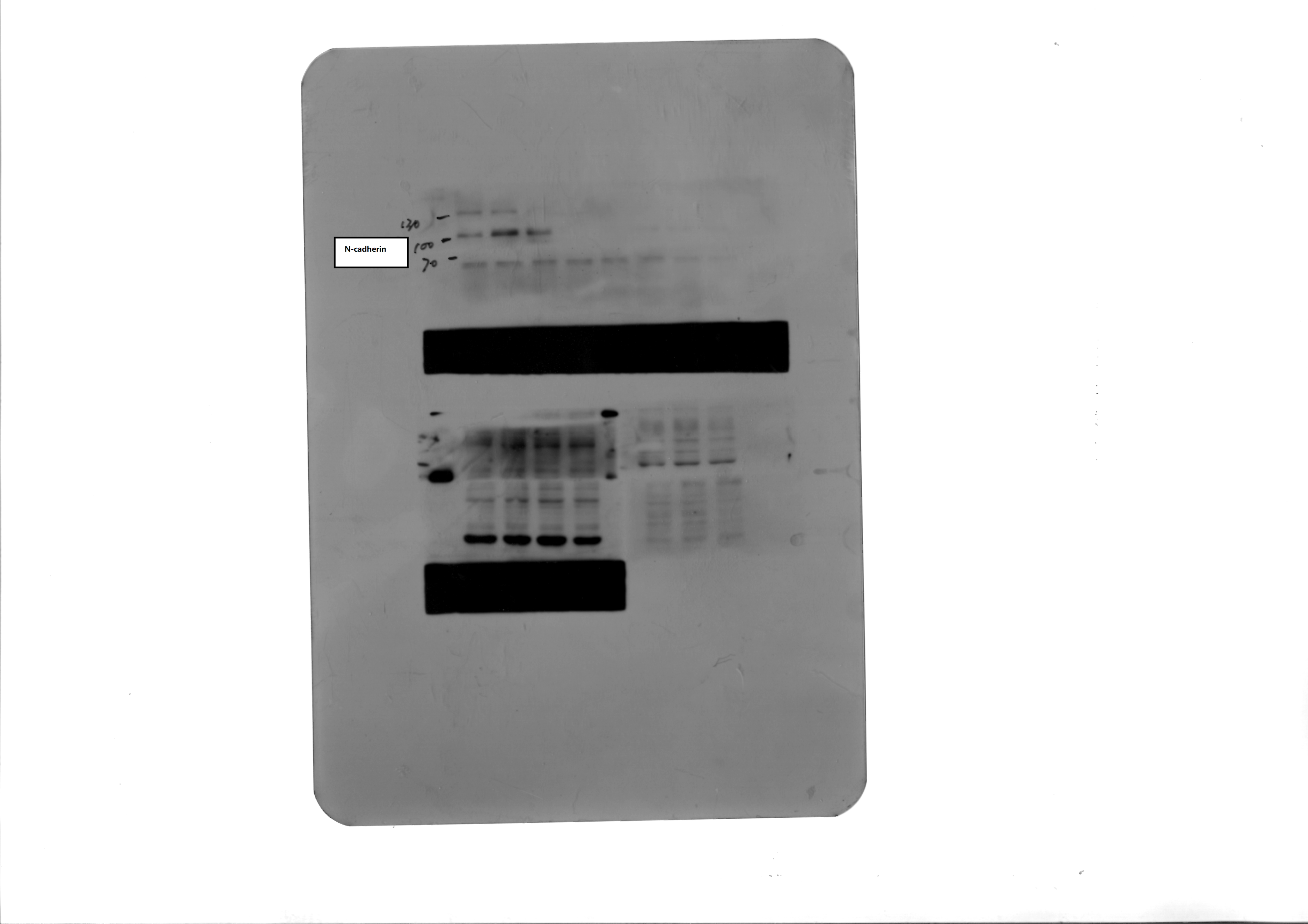

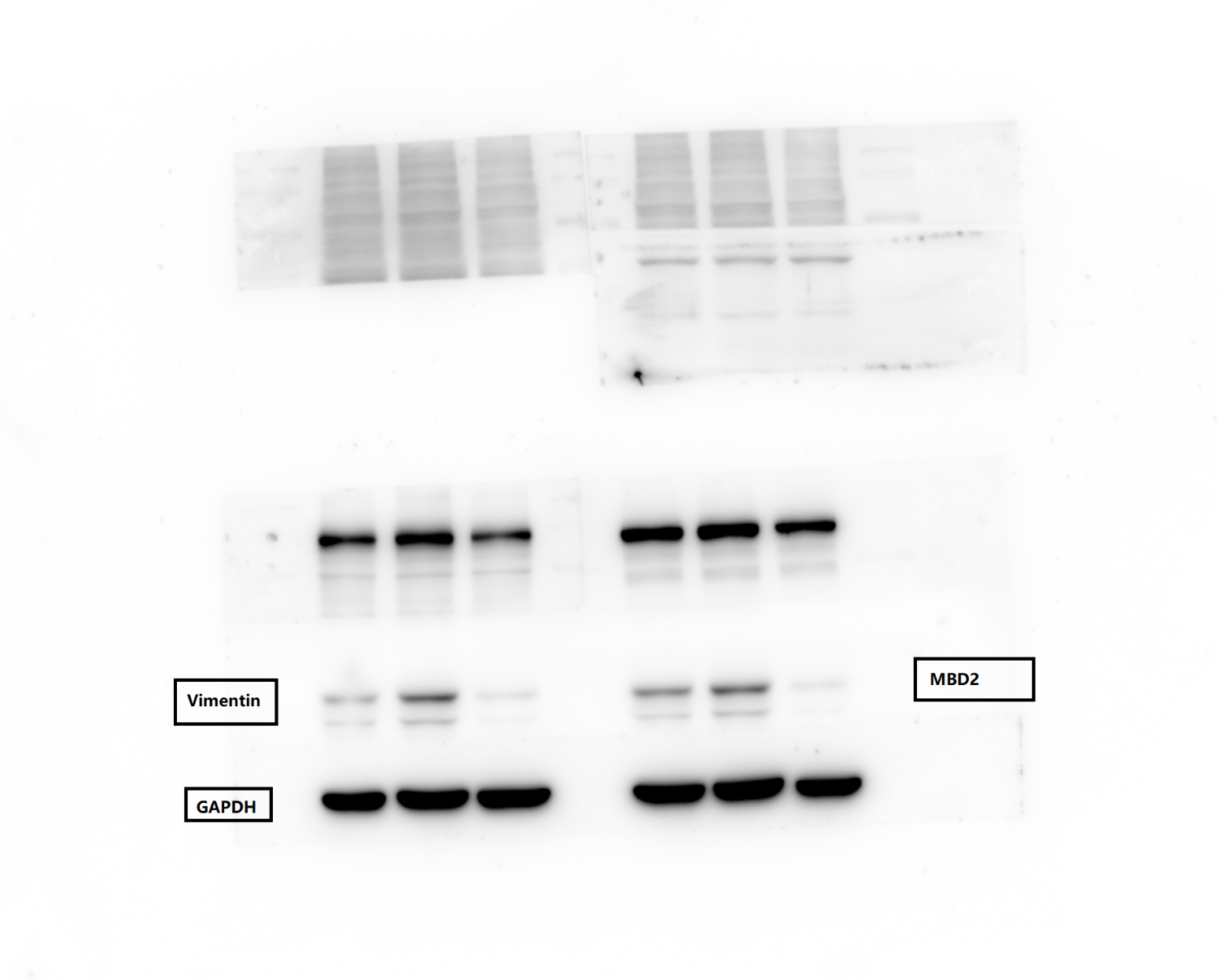

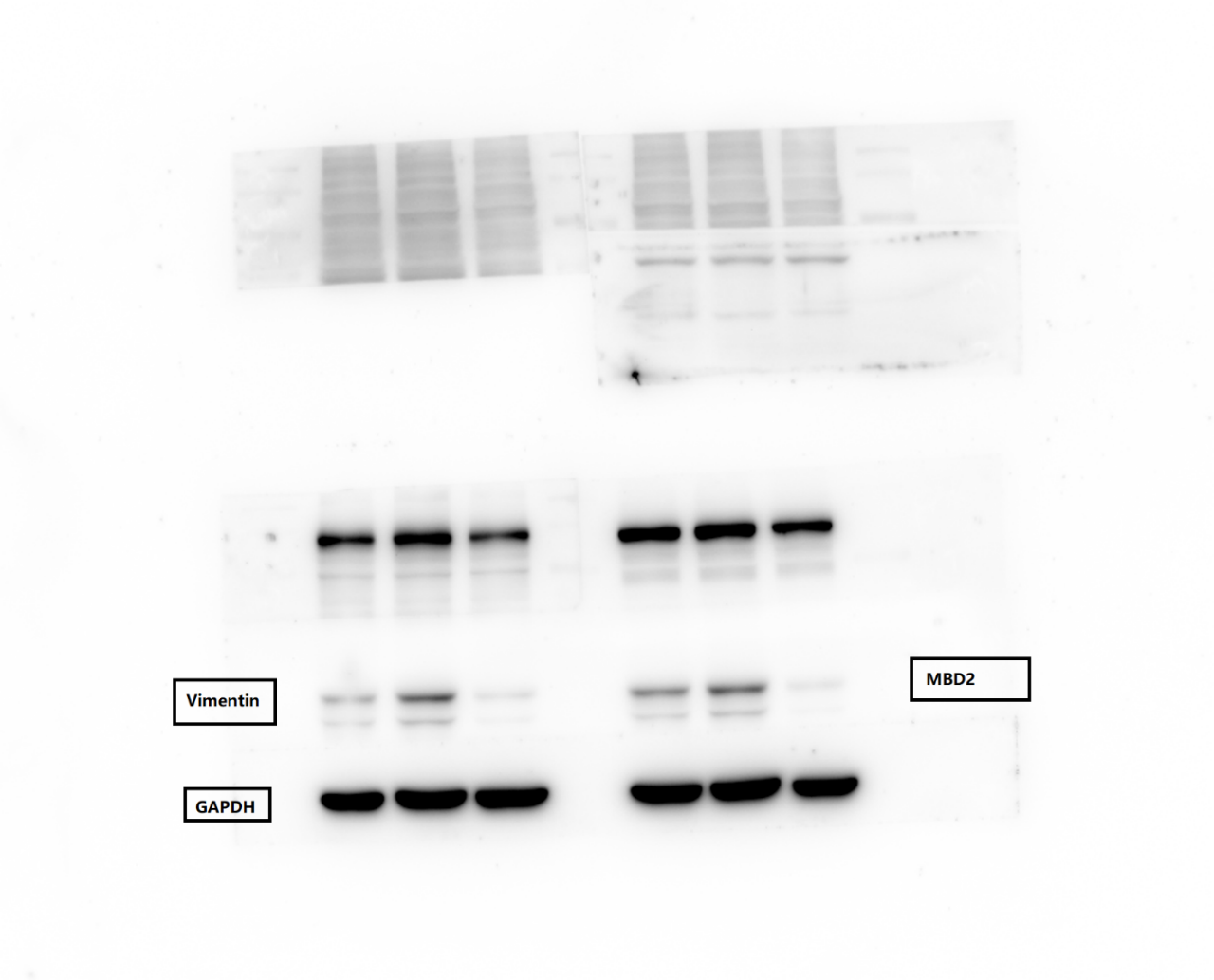


Figure 2F：


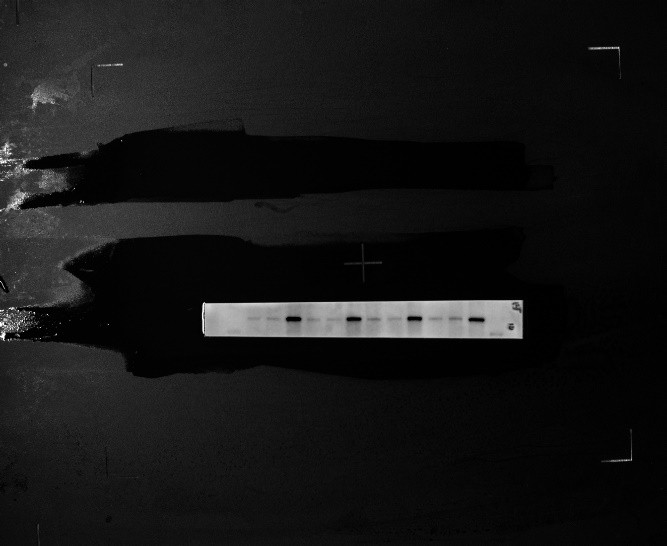

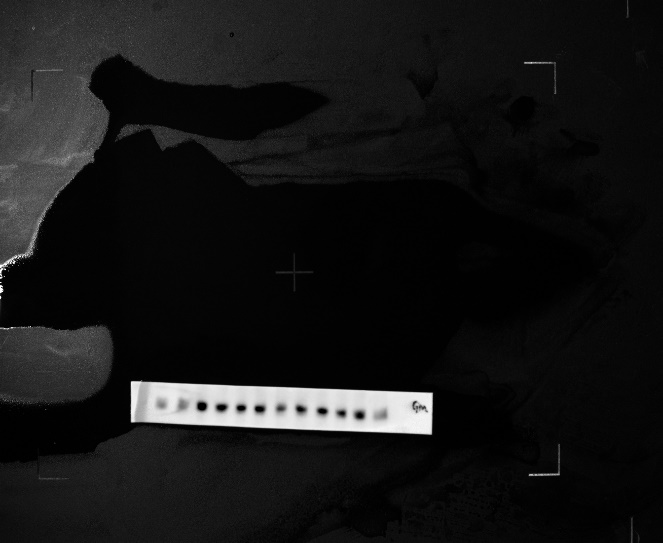

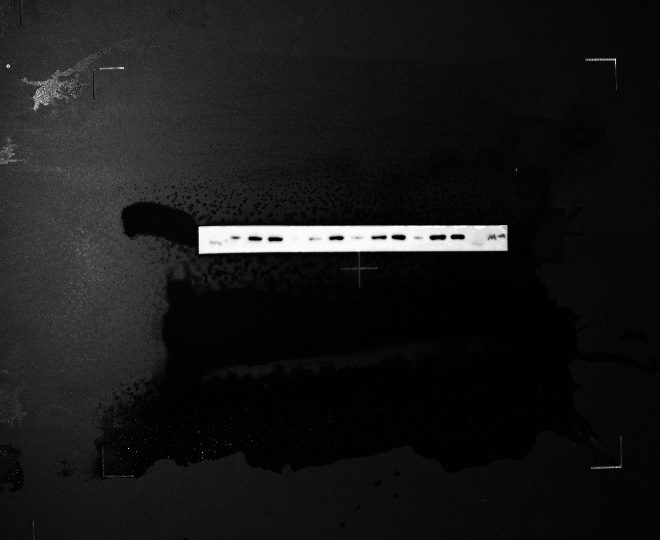

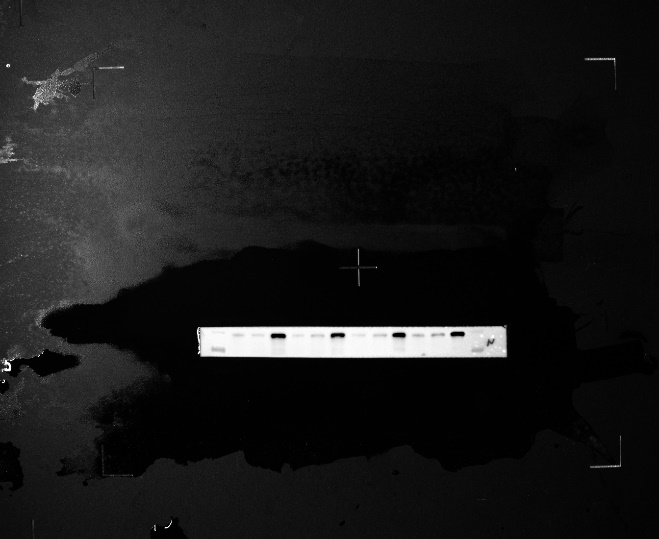


B16F10 N-cadherin

B16F10 MBD2

B16F10 GAPDH

B16F10 E-cadherin

Figure 4C (A549 cells):
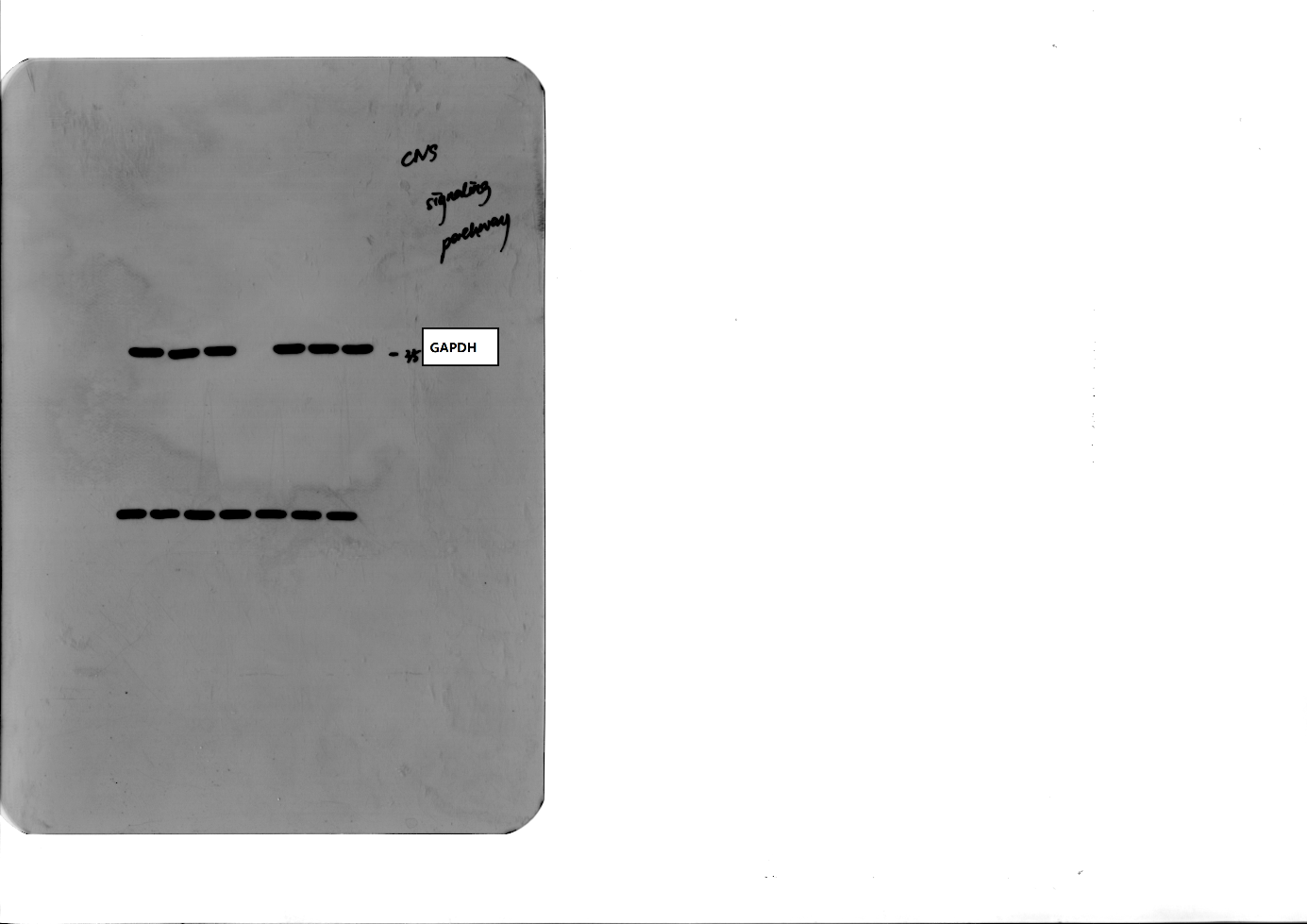




MBD2

Figure 4D (B16F10 cells)：


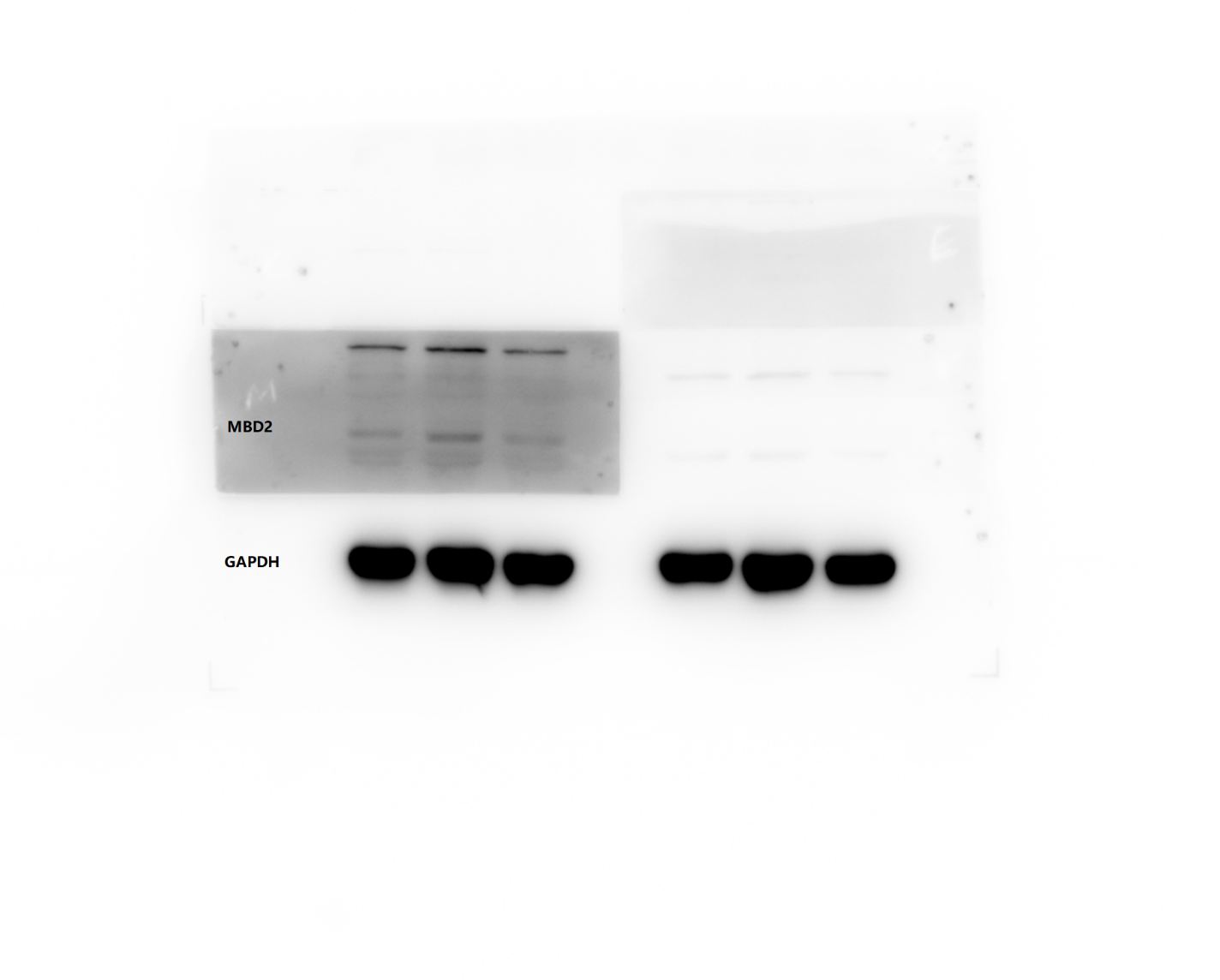


Figure 5D：


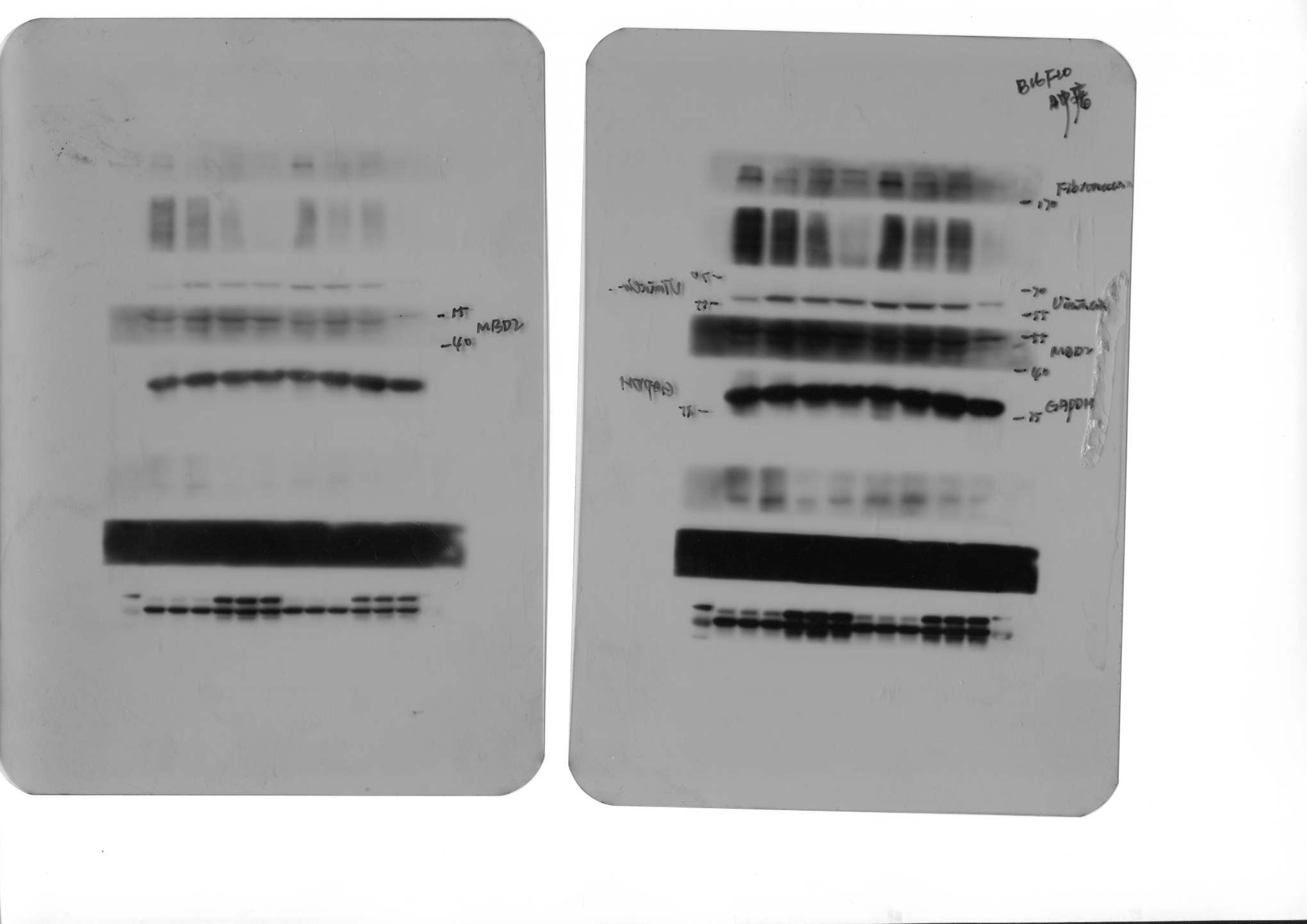

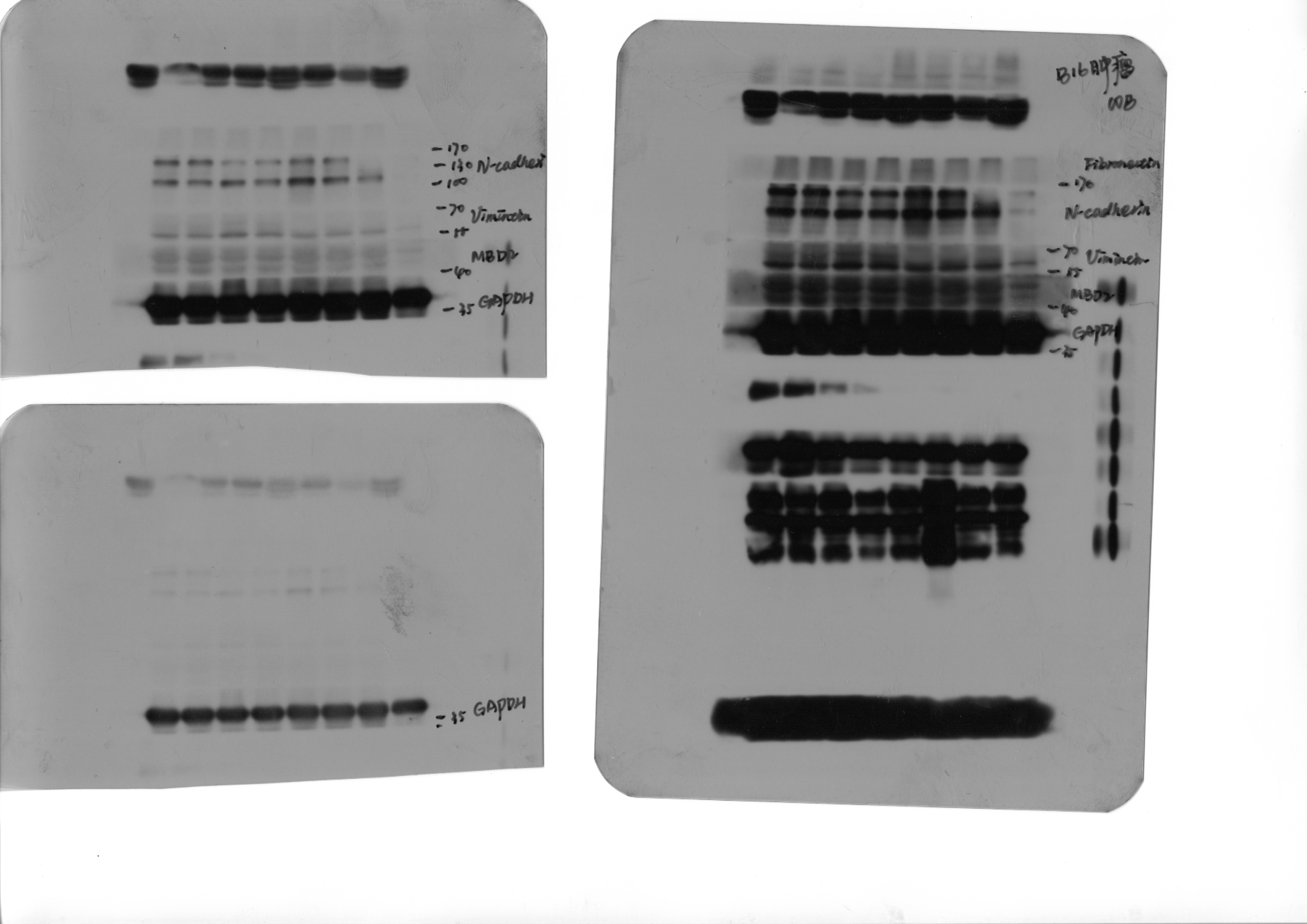


GAPDH

MBD2

Supplementary Fig. 2A


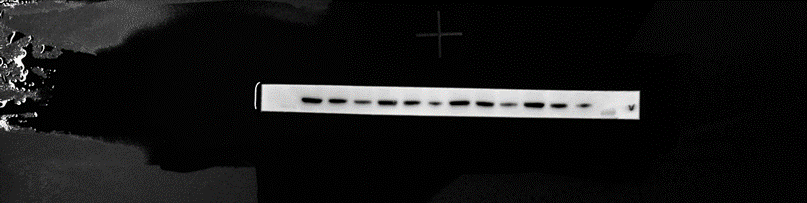


A549 E-cadherin


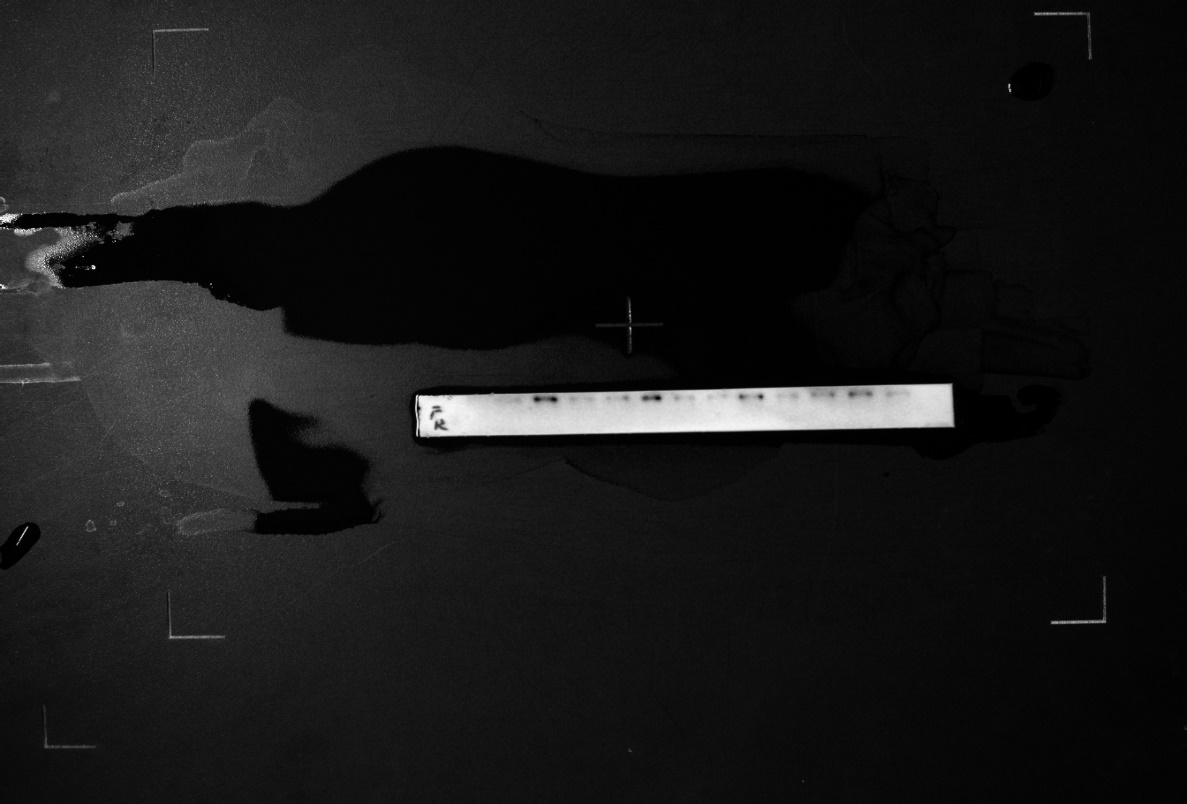


A549 N-cadherin


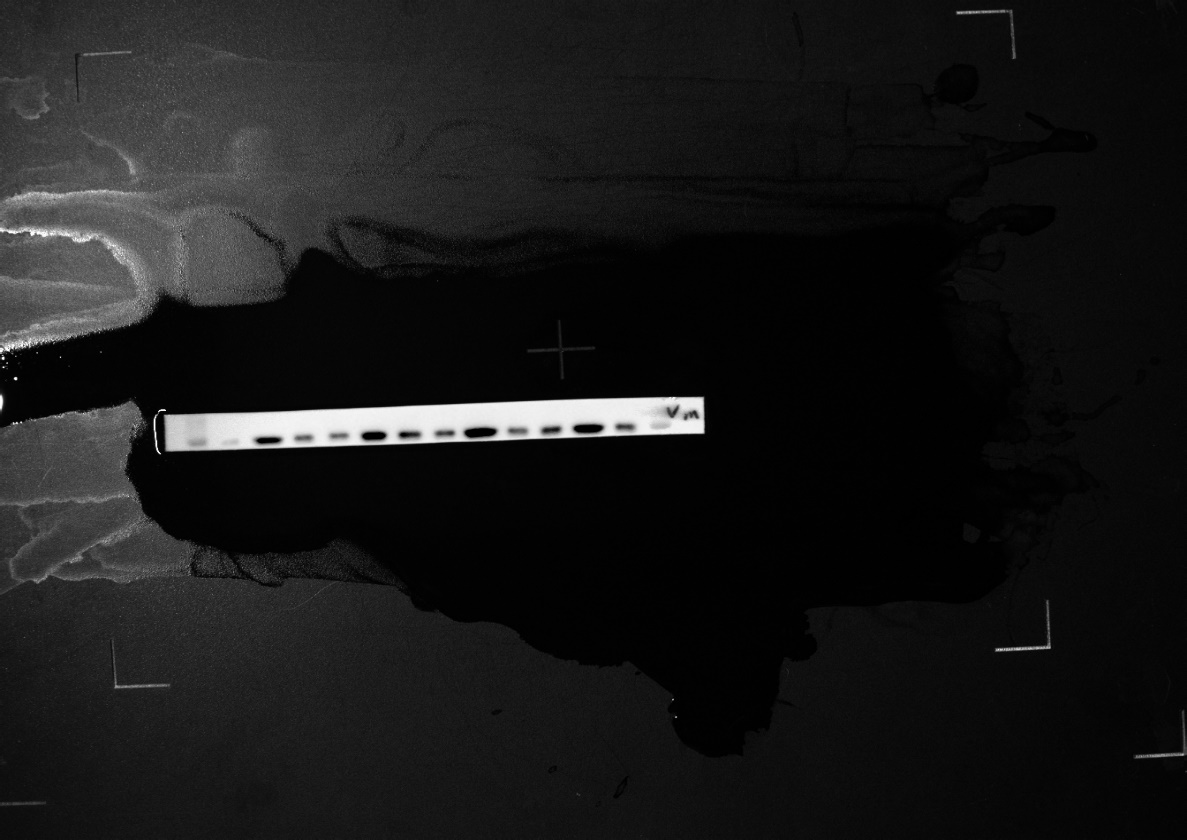


A549 Vimentin


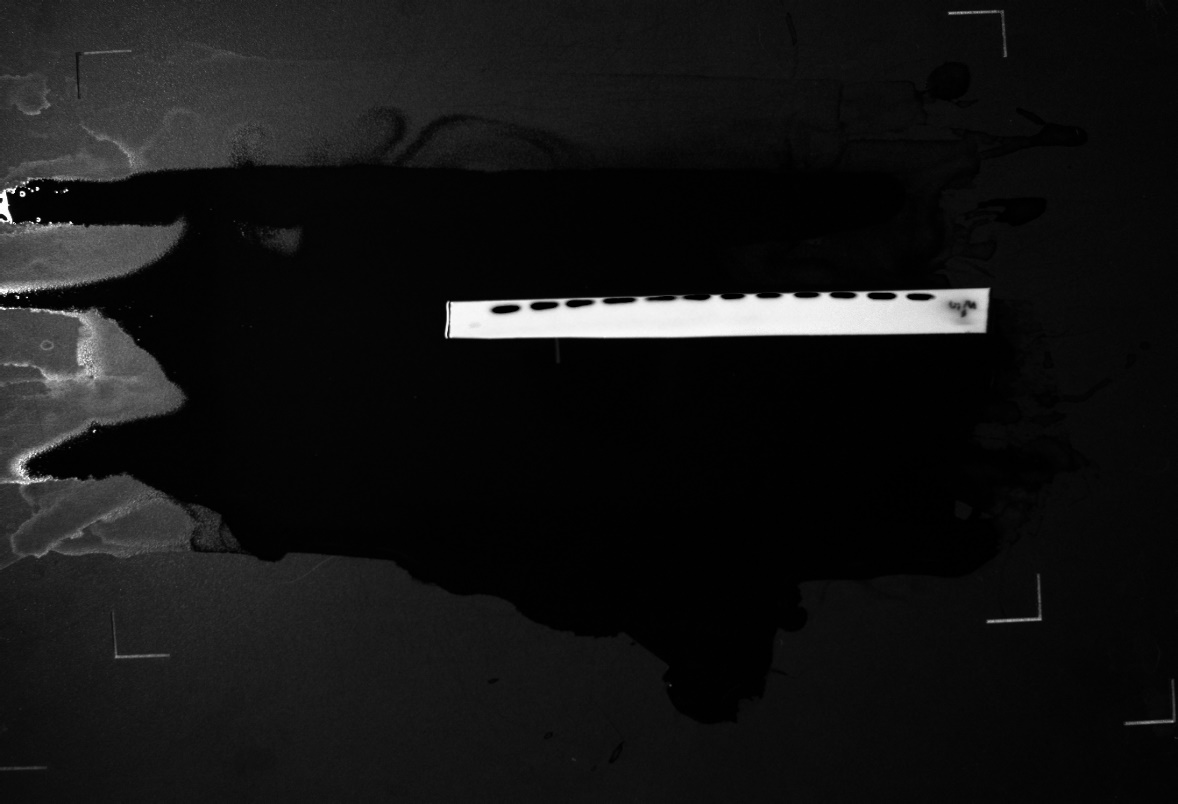


A549 GAPDH


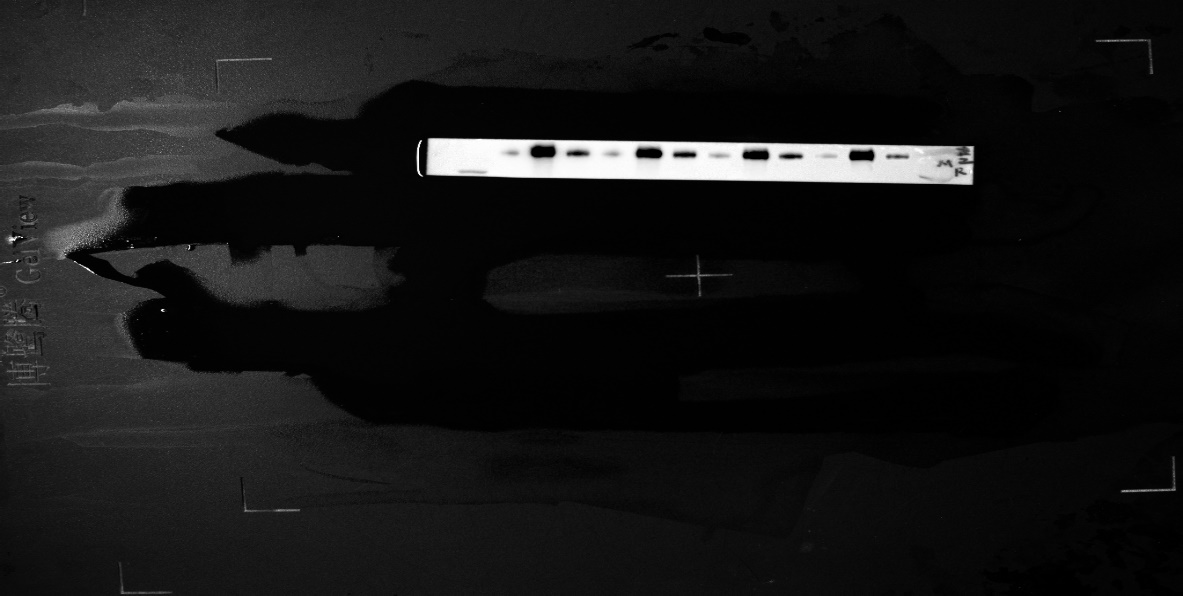


A549 MBD2

Supplementary Fig. 2C


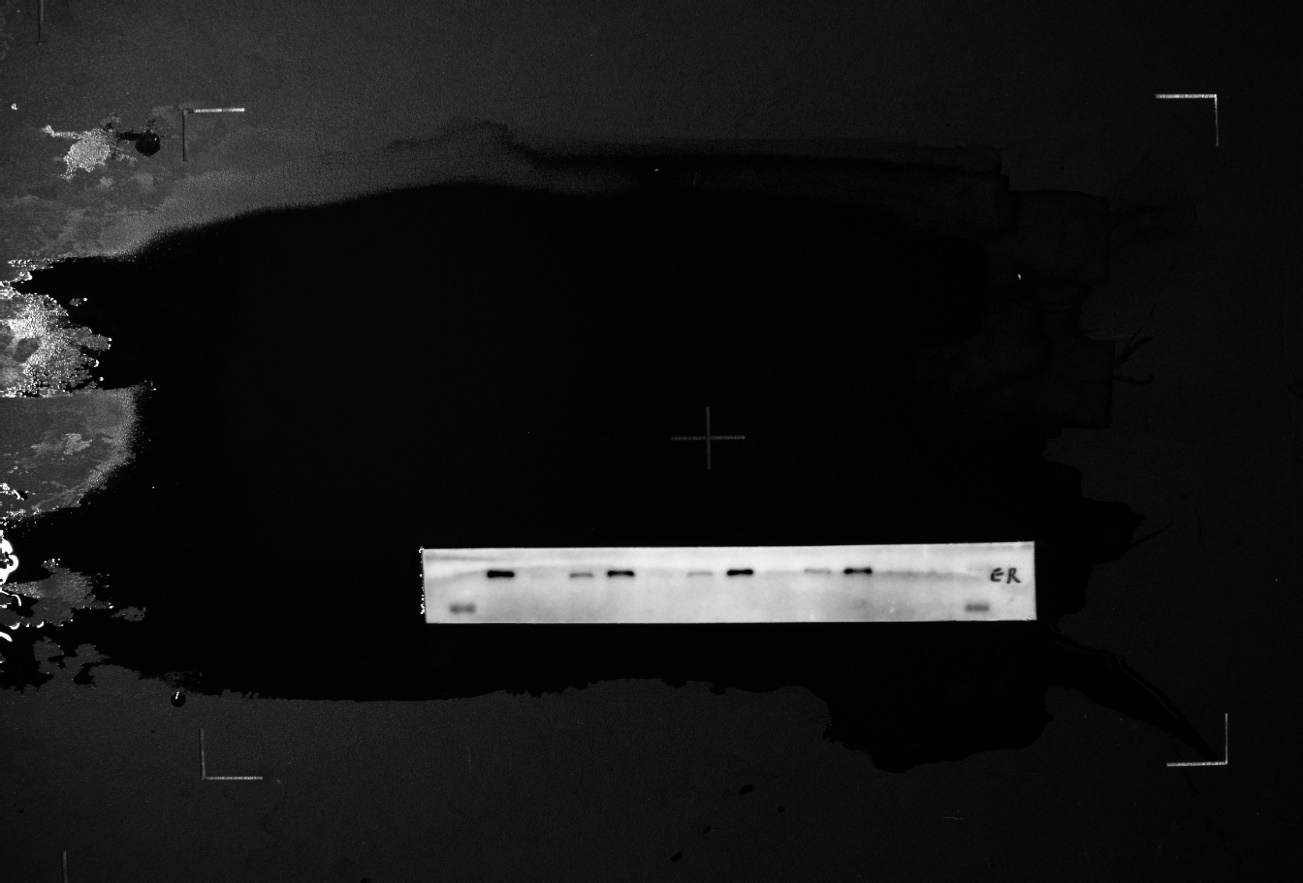


B16F10 E-cadherin


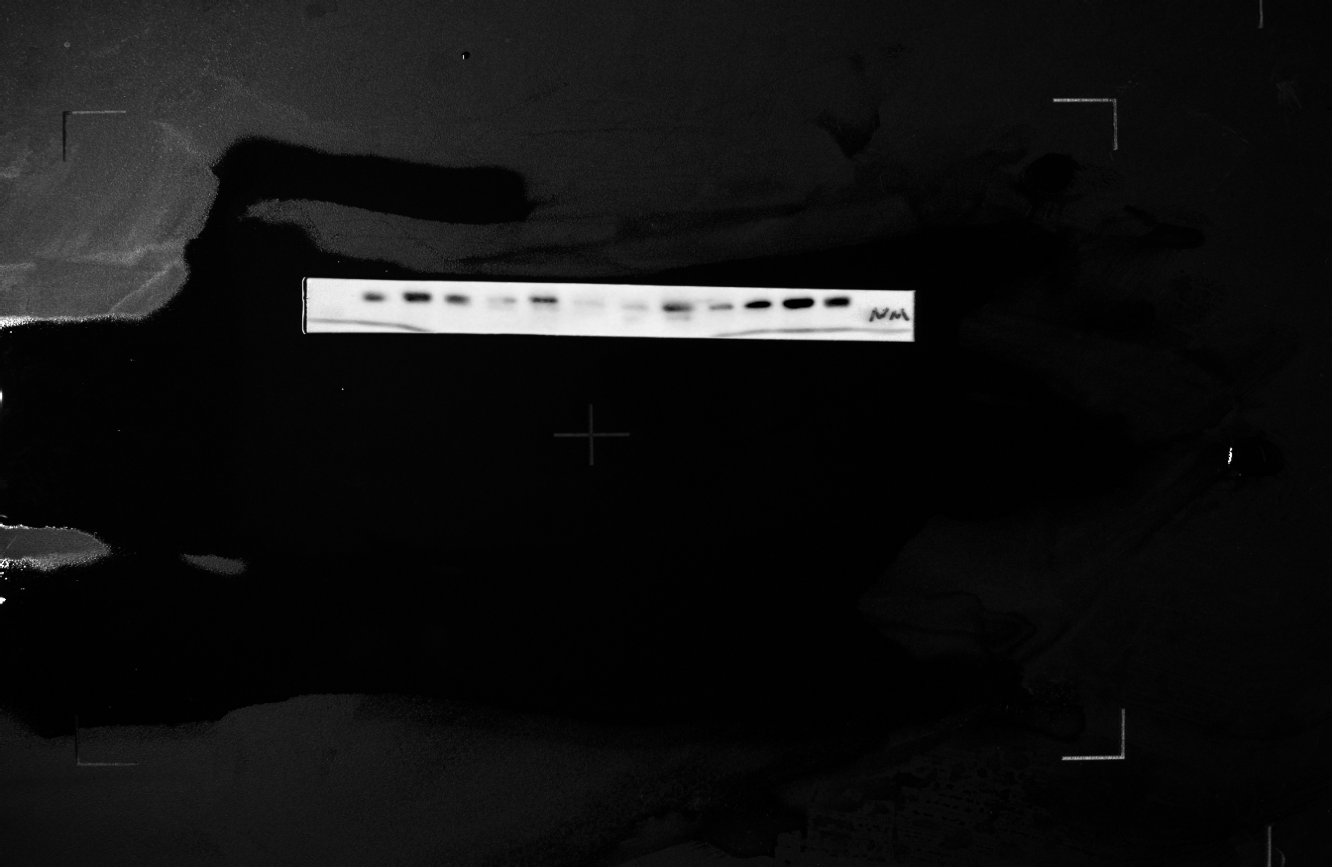


B16F10 N-cadherin


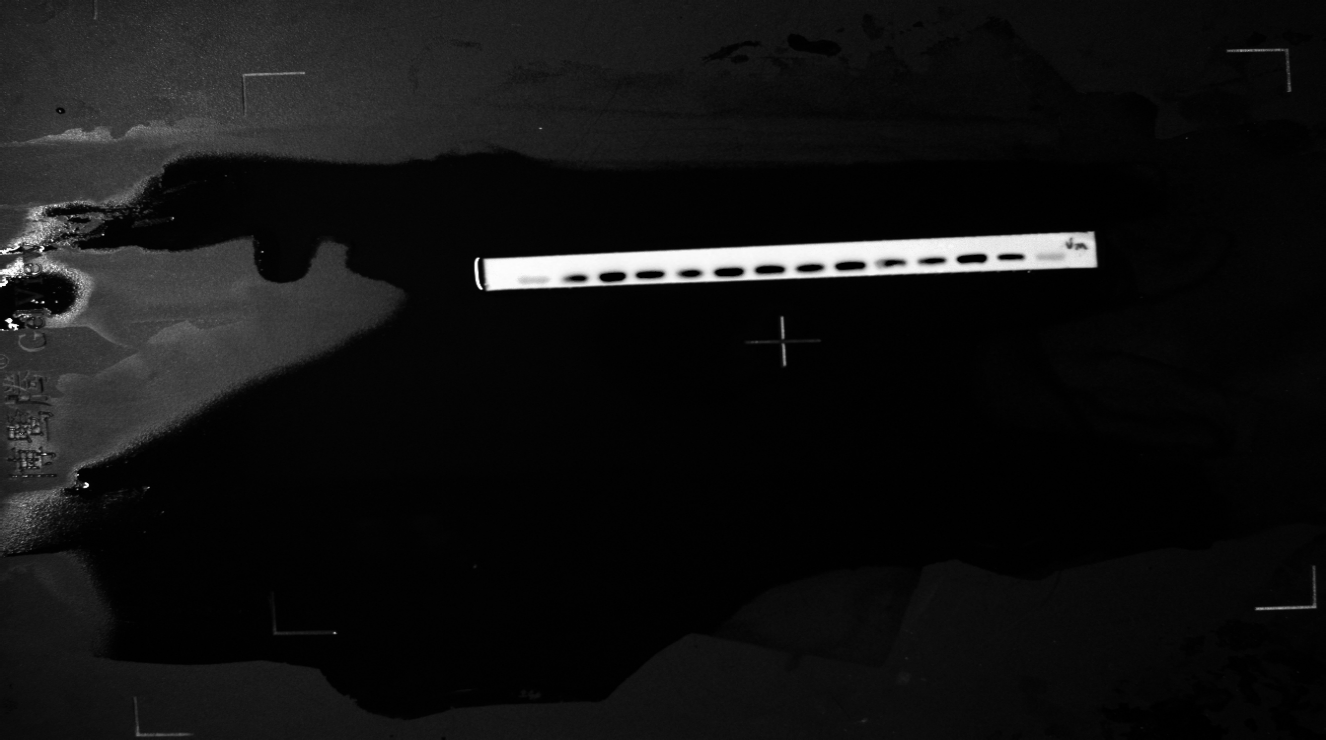


B16F10 Vimentin


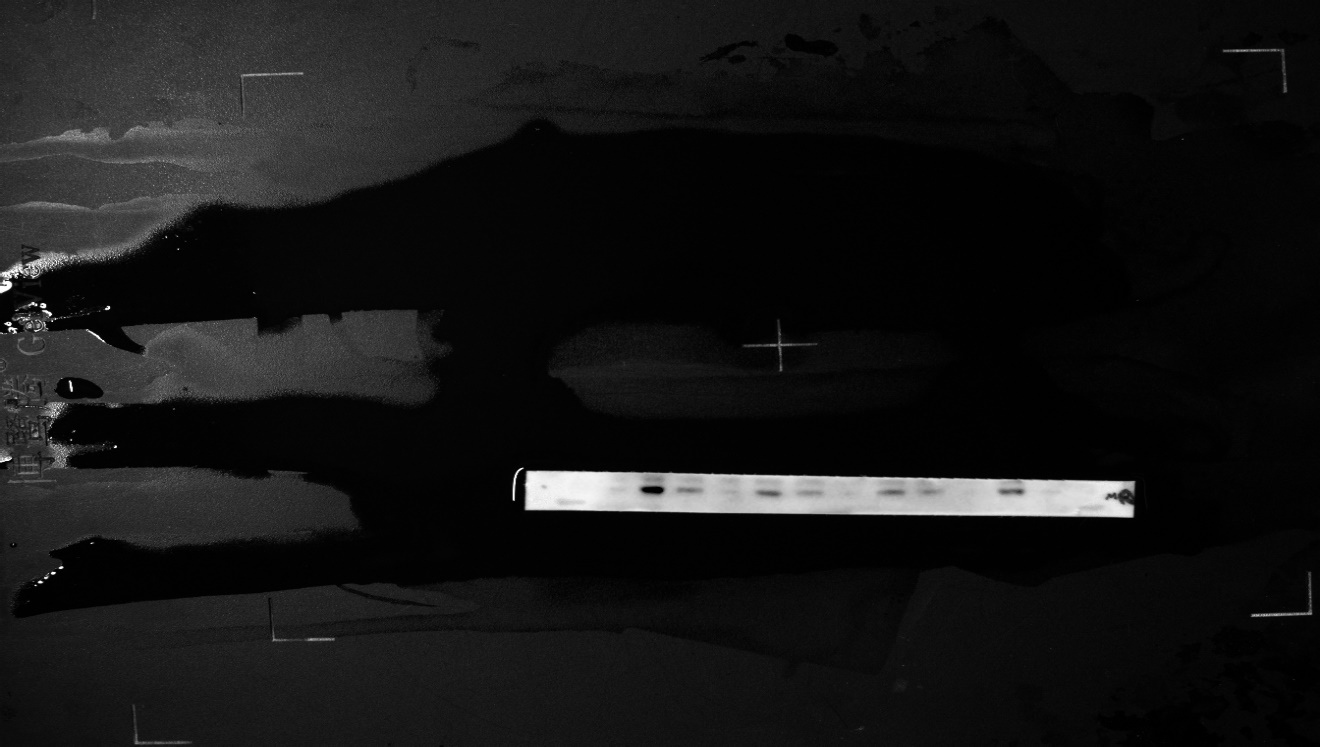


B16F10 MBD2


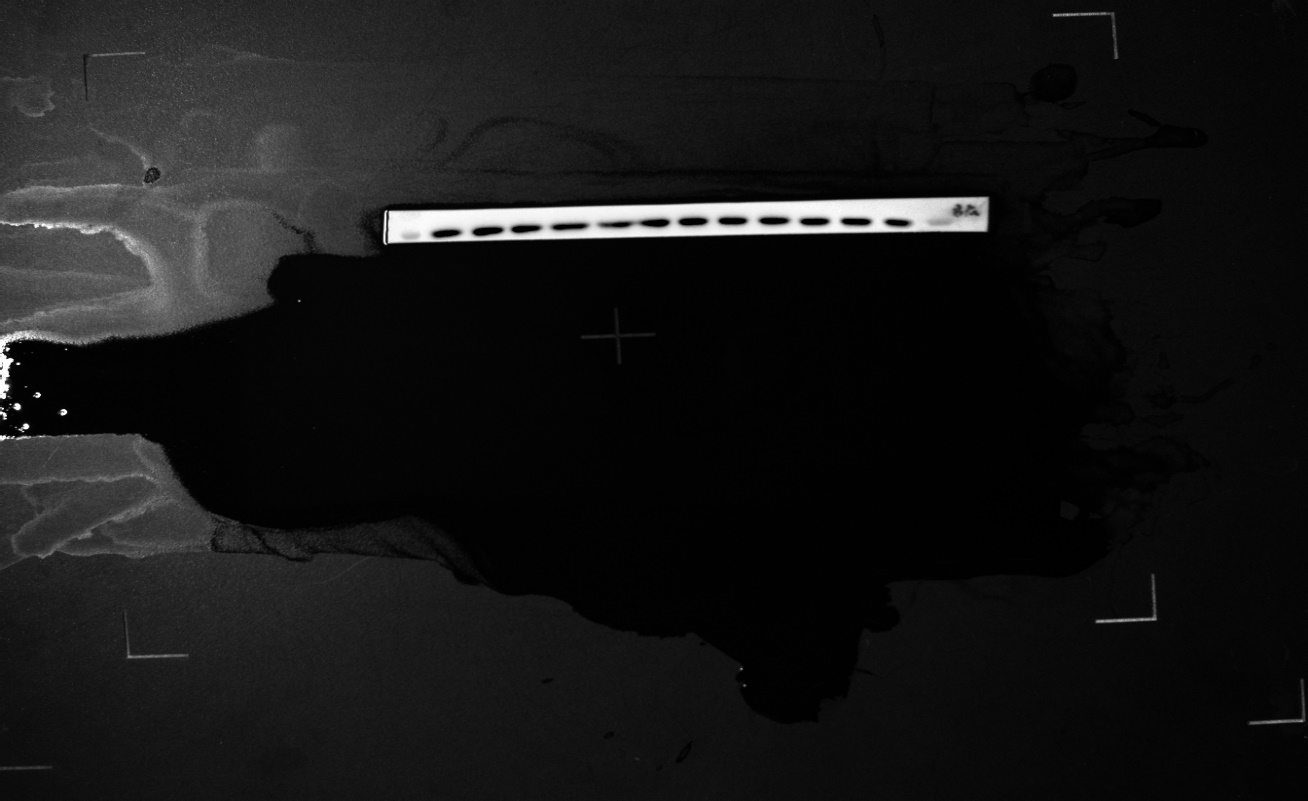


B16F10 GAPDH

Supplementary Fig. 2E


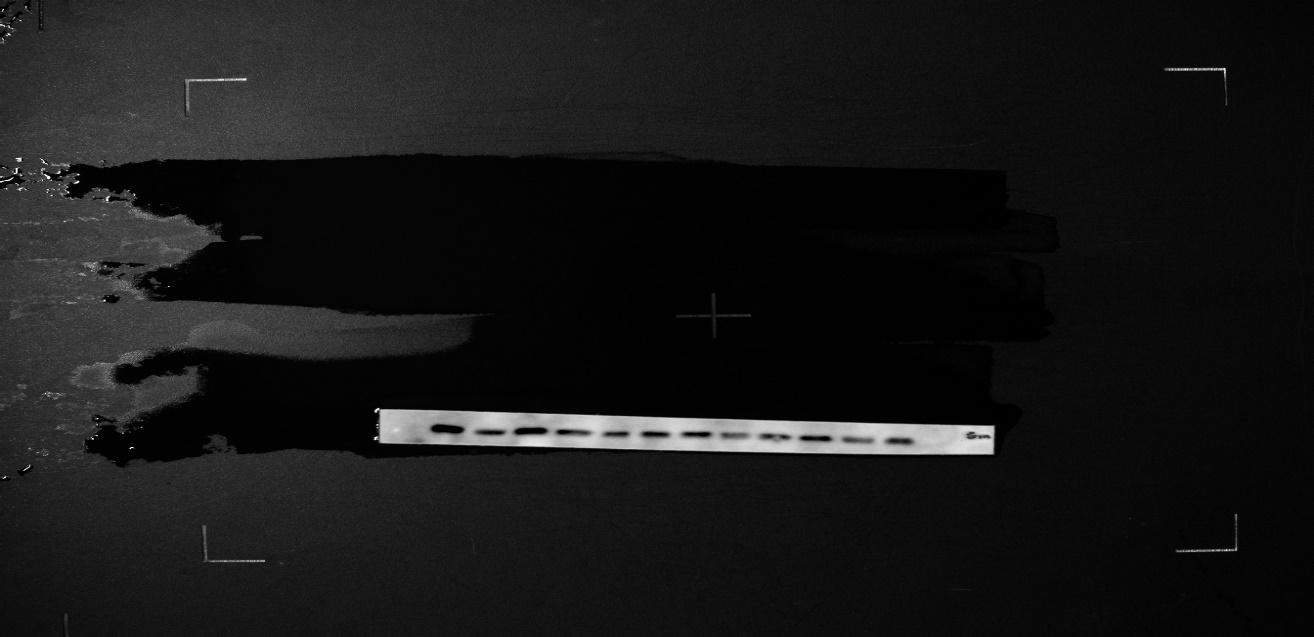


H1975 E-cadherin


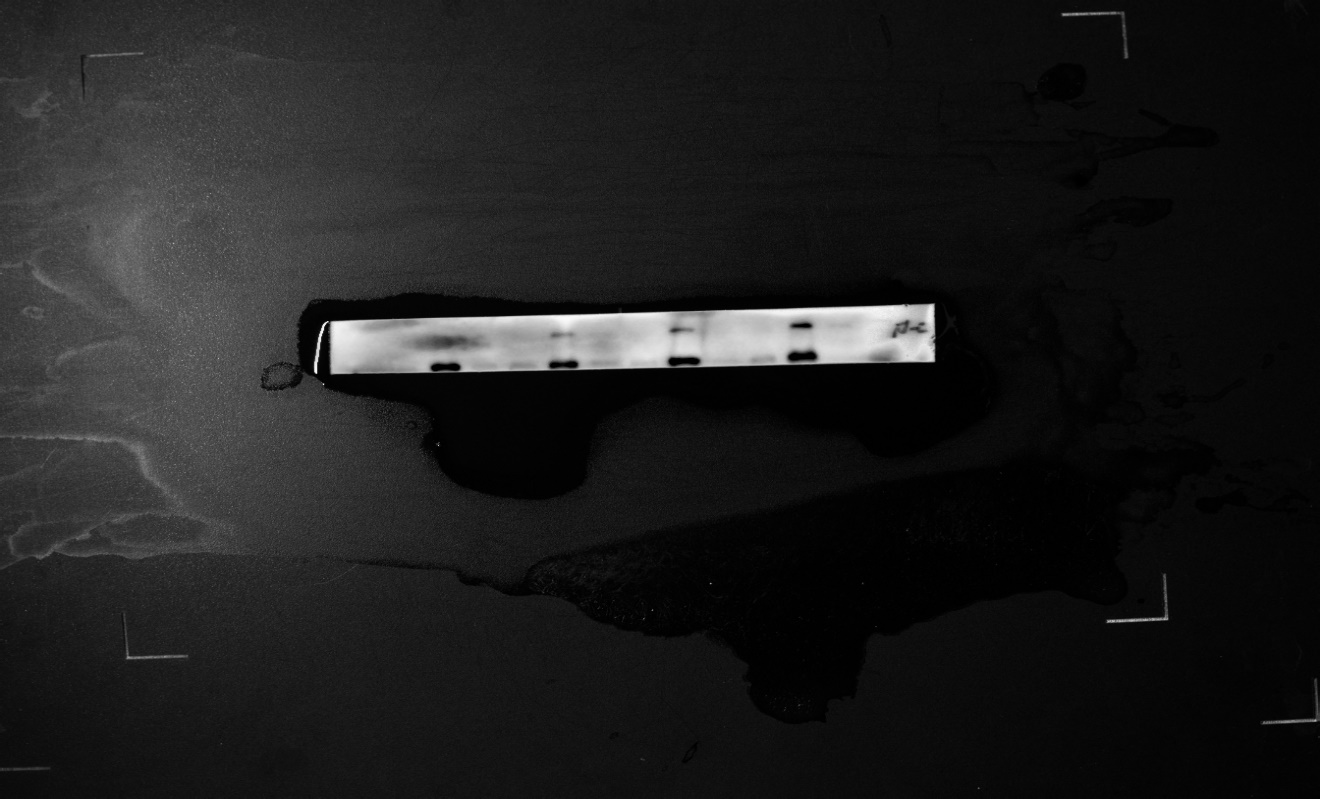


H1975 N-cadherin


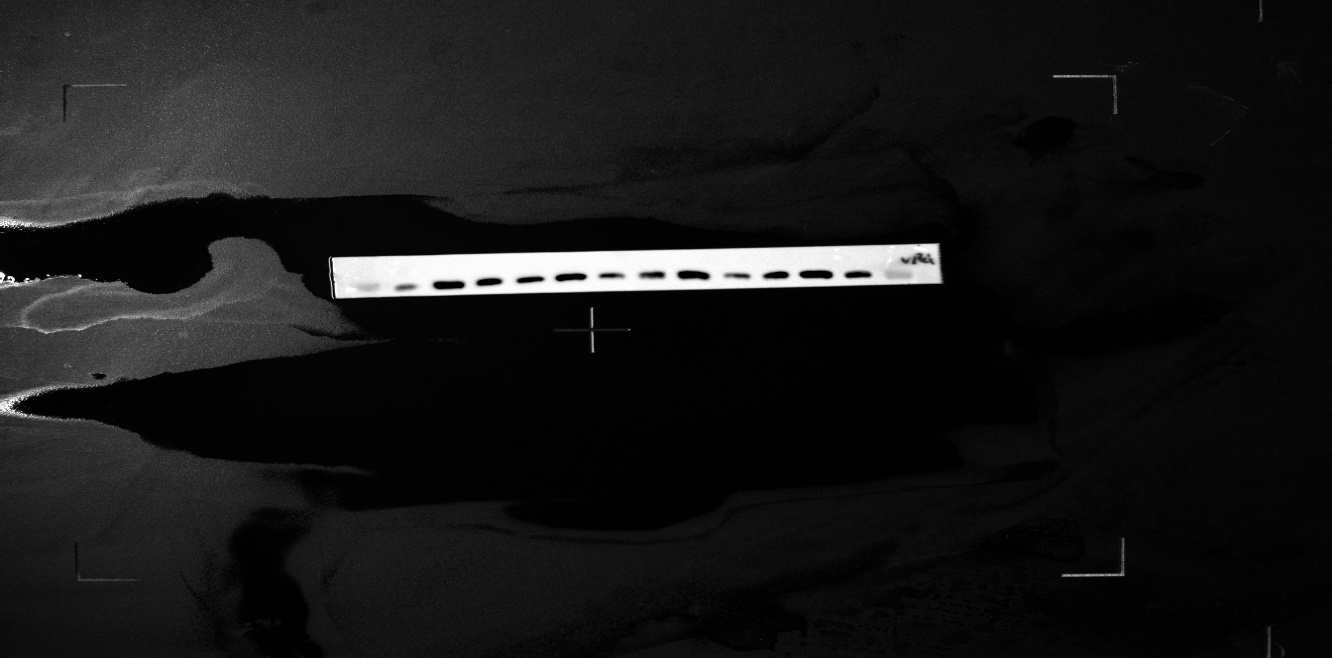


H1975 Vimentin


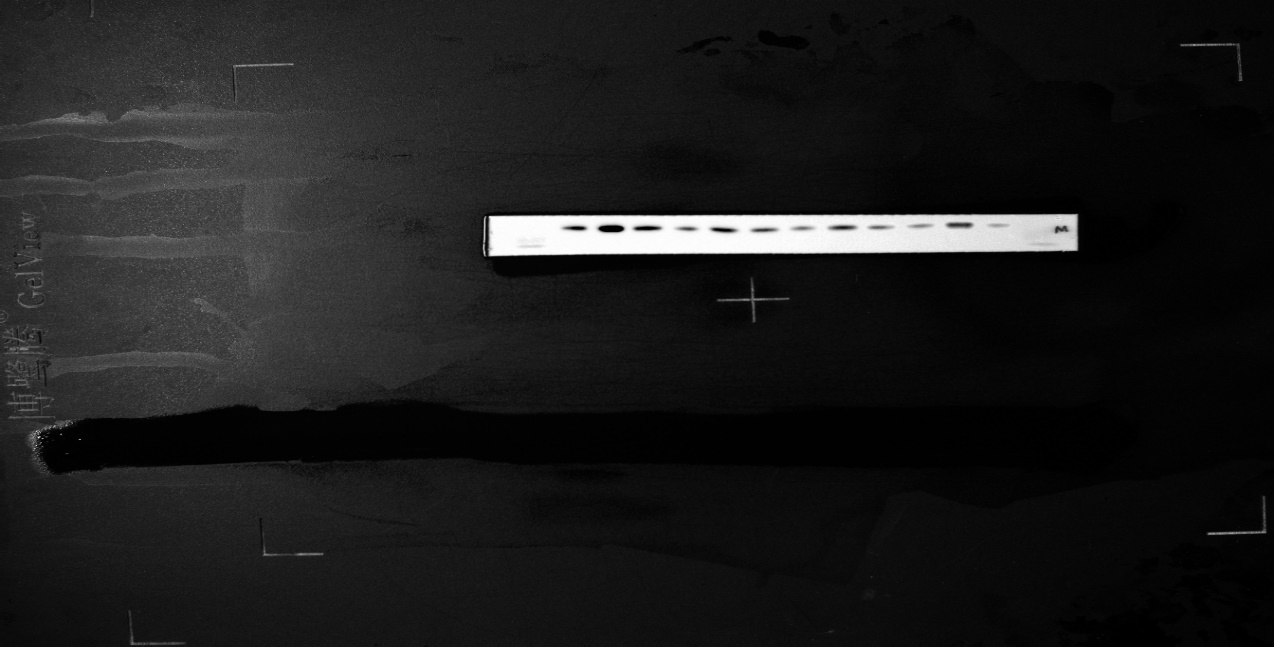


H1975 MBD2


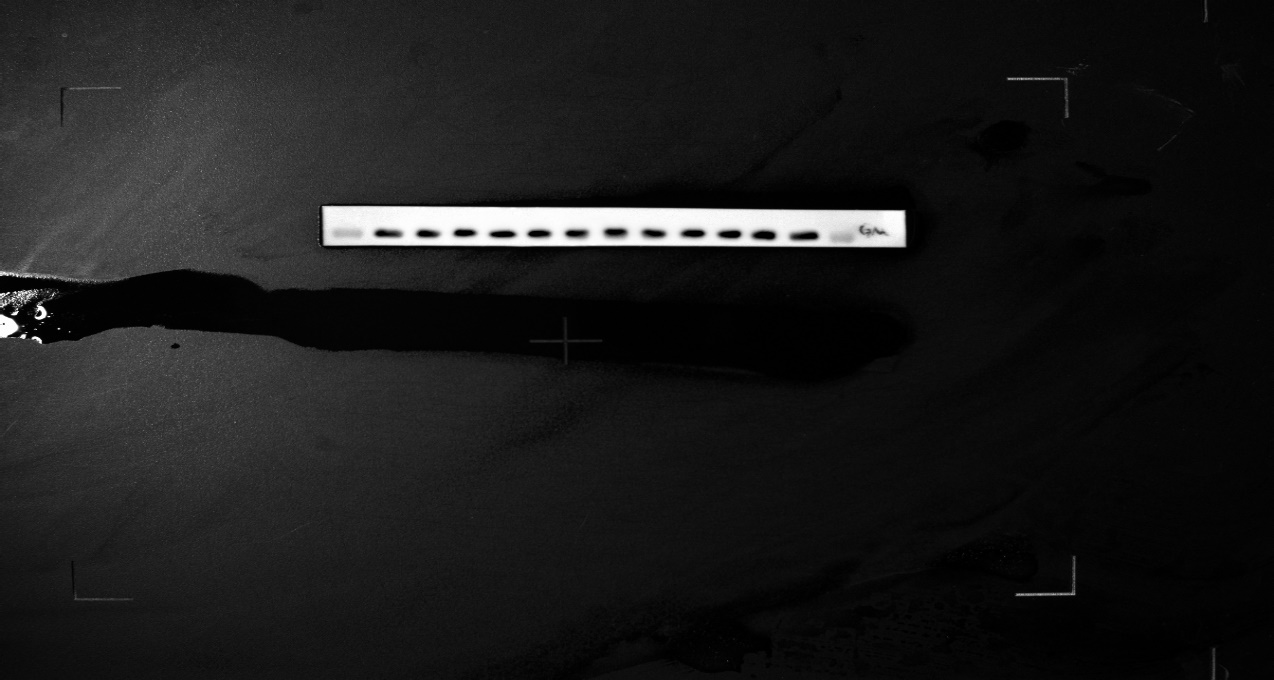


H1975 GAPDH
